# Associations of Outdoor Air Pollution With Incidence of Cancers Other Than Lung Cancer in a Large US Prospective Cohort

**DOI:** 10.1002/ijc.70530

**Published:** 2026-05-03

**Authors:** W. Ryan Diver, Lauren R. Teras, Emily L. Deubler, Alpa V. Patel, Michelle C. Turner

**Affiliations:** ^1^ Barcelona Institute for Global Health (ISGlobal) Barcelona Spain; ^2^ Universitat Pompeu Fabra (UPF) Barcelona Spain; ^3^ Department of Population Science American Cancer Society Atlanta Georgia USA; ^4^ CIBER Epidemiología y Salud Pública (CIBERESP) Madrid Spain

**Keywords:** cancer incidence, nitrogen dioxide, outdoor air pollution, particulate matter, prospective cohort

## Abstract

Outdoor air pollution, including particulate matter (PM), is a Group 1 carcinogen based on evidence for lung cancer; however, evidence for other cancers is limited. Further research on cancer incidence rather than mortality endpoints is needed as well as examinations in non‐smokers. In the Cancer Prevention Study‐II Nutrition Cohort, annual predictions of particulate and gaseous pollutant concentrations were assigned to residential addresses. Extended Cox regression estimated hazard ratios (HR) and 95% confidence intervals (CI) for associations of pre‐diagnosis pollutants with risk of all incident non‐lung cancers and 20 cancer sites with detailed adjustment for confounding variables. There were 28,008 incident cancers identified among the 108,002 participants followed from 1992 to 2017. There were no statistically significant associations with fine particulates (PM_2.5_) overall or among never smokers. There were elevated HRs with coarse particulates (PM_10‐2.5_) for uterine and cervical cancer incidence, though CIs were wide, and an association with ER‐ breast cancers (HR = 1.16; 95% CI 1.03–1.30). There were also weak positive associations of gaseous pollutants in never smokers with colorectal cancer (sulfur dioxide HR = 1.08; 95% CI 1.01–1.16), kidney cancer (carbon monoxide HR = 1.17; 95% CI 1.00–1.37), and melanoma of the skin (ozone HR = 1.11; 95% CI 0.99–1.25). Overall, these findings indicate few positive associations of ambient air pollutants with cancer incidence beyond lung cancer. The observed associations were low magnitude and stronger in never smokers. Larger pooled studies are needed to validate these associations with rare subtypes and non‐smokers, and cancer survival research is needed to clarify differences in mortality and incidence studies.

## Abstract (highlights)

What's new?

Associations between outdoor air pollution and cancers other than lung cancer remain unclear, particularly in the context of tobacco use. Here, the authors assessed possible links between ambient air pollutant exposure and incidence of 20 non‐lung cancers among adults followed over a 25‐year period in the U.S. Cancer Prevention Study‐II Nutrition Cohort. Few associations were identified overall. Among never smokers, modest links were observed between certain gaseous pollutants and melanoma, colorectal cancer, and kidney cancer. The results indicate that air pollution has limited carcinogenic effects beyond lung cancer overall, though specific associations, particularly in never smokers, deserve further study.

## Graphical abstract

Associations between outdoor air pollution and cancers other than lung cancer remain unclear, particularly in the context of tobacco use. Here, the authors assessed possible links between ambient air pollutant exposure and incidence of 20 non‐lung cancers among adults followed over a 25‐year period in the United States. Cancer Prevention Study‐II Nutrition Cohort. Few associations were identified overall. Among never smokers, modest links were observed between certain gaseous pollutants and melanoma, colorectal cancer, and kidney cancer. The results indicate that air pollution has limited carcinogenic effects beyond lung cancer overall, though specific associations, particularly in never smokers, deserve further study.

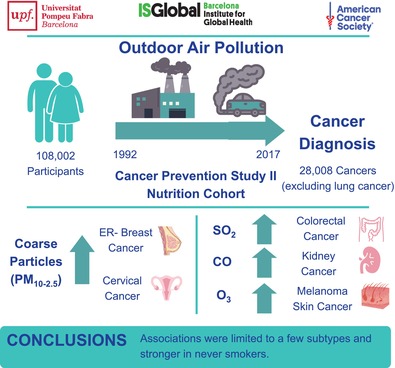

**Abbreviations**
BMI: body mass index
CACES: Center for Air, Climate, and Energy Solutions
CI: confidence interval
CO: carbon monoxide
CPS‐II: Cancer Prevention Study‐II
ER: estrogen receptor positive (+) and negative (−)
HR: hazard ratio
IARC: International Agency for Research on Cancer
NDI: National Death Index
NO_2_
: nitrogen dioxide
O_3_
: ozone
PAH: polycyclic aromatic hydrocarbons
PM_10_
: particulates with an aerodynamic diameter ≤ 10 μm
PM_10‐2.5_
: particulates with an aerodynamic diameter ≤ 10 and > 2.5 μm
PM_2.5_
: particulates with an aerodynamic diameter ≤ 2.5 μm
PSA: prostate‐specific antigen
RR: rate ratio
SO_2_
: sulfur dioxide

## 1 Introduction

Outdoor air pollution and particulate matter (PM) in outdoor air have been defined as carcinogenic to humans (Group 1) by a Working Group convened by the International Agency for Research on Cancer (IARC) [1]. The human evidence for the classification was primarily related to associations with PM_2.5_ and lung cancer mortality [2, 3, 4]. The Working Group noted that there were also positive associations of outdoor air pollution with bladder cancer; however, information was insufficient to determine carcinogenicity due to few studies and small sample sizes. Since the IARC evaluation in 2013, additional evidence related to lung cancer incidence has continued to confirm the findings of the evaluation [5, 6].

Beyond lung cancer, there have been studies on outdoor air pollution and cancer with a growing body of research at several cancer sites [7]. Meta‐analyses have shown no overall association with PM_2.5_ or PM_10_ and breast cancer incidence, although there has been some indication of stronger findings in Europe than North America and by hormone receptor status [8, 9]. Another meta‐analysis reported a positive though weak association of nitrogen dioxide (NO_2_) with breast cancer (RR per 10 μg/m^3^ = 1.015; 95% CI 1.003–1.028) [10]. Studies on gastrointestinal cancers were reviewed with strongest findings observed for PM_2.5_ with colorectal (HR per 10 μg/m^3^ = 1.35; 95% CI 1.08–1.62) and liver cancer (HR per 10 μg/m^3^ = 1.31; 95% CI 1.07–1.56) [11]. However, there was high heterogeneity among the included studies suggesting that methodological differences may play a role. Some positive associations have been observed with bladder and kidney cancer but larger studies with longer follow‐up are needed [12]. Importantly, much of the previous literature has limited data on tobacco smoking which may substantially confound studies of air pollution.

In the American Cancer Society Cancer Prevention Study‐II (CPS‐II), 623,048 participants were followed for 22 years (1982–2004) for cancer mortality with a total of 43,320 non‐lung cancer deaths observed [13]. For every 4.4 μg/m^3^ increase in PM_2.5_ there was an increase in risk of kidney (HR = 1.14; 95% CI 1.03–1.27) and bladder cancer (HR = 1.13; 95% CI 1.03–1.23) mortality, as well as an increase in colorectal cancer mortality associated with each 6.5 ppb increase in NO_2_ (HR = 1.06; 95% CI 1.02–1.10). However, fatal cancers constitute a select group that do not represent most cancer diagnoses and research on incident disease is needed to understand the true impacts associated with ambient air pollution. CPS‐II research on air pollution and incident lung cancer focused on the less studied topics of subtypes and survival after lung cancer diagnosis; however, research on incidence at other cancer sites has not been conducted [14].

In this study, we are using a subset of the larger CPS‐II mortality study that is followed for incident cancer to examine whether ambient air pollution is associated with risk of a first cancer diagnosis at sites other than the lung. With 25 years of follow‐up in a large US population of over 100,000 participants, updated residential address and pollutant data during follow‐up, and detailed cancer incidence information, this study addresses the need for additional studies on incidence of specific cancer sites, including greater numbers of common less fatal cancers than previous work based on cancer mortality. Additionally, analysis of over 46,000 participants with no smoking history addresses concerns about residual confounding by tobacco use.

## 2 Materials and Methods

### 2.1 Study Population

Participants were selected from the CPS‐II Nutrition Cohort, a US prospective study of cancer incidence and mortality in 184,183 men and women, described in Calle et al. [15] In brief, the Nutrition Cohort is a sub‐cohort of the larger CPS‐II mortality cohort, a prospective study of ~1.2 million participants established by the American Cancer Society in 1982. In the CPS‐II mortality cohort, participants were recruited nation‐wide and completed a questionnaire at enrollment which included residential address information. The Nutrition Cohort includes CPS‐II participants from 21 states with population‐based state cancer registries who were invited to participate in the sub‐cohort in 1992. A mailed questionnaire was completed by participants that included information on demographic, medical, behavioral, environmental, occupational, and dietary factors. Questionnaires were sent to the cohort members every 2 years beginning in 1997 and continuing through 2017 to update risk factor data, identify new cancer diagnoses, and update residential address information. Follow‐up surveys were received from at least 87% of living participants after multiple mailings.

Participants were excluded from the analysis if they did not return any survey after baseline in 1992/1993 and did not die prior to the first follow‐up survey (i.e., lost to follow‐up) (*n* = 6190), reported a personal history of cancer other than non‐melanoma skin cancer at baseline in 1992/1993 (*n* = 22,870), had poor quality address linkage (*n* = 41,225), their address included a PO Box or “Care of” (*n* = 5383), or reported a cancer diagnosis on the first follow‐up survey which could not be verified (*n* = 513). The final analytic cohort included 108,002 men and women (Figure S1).

### 2.2 Exposures

We obtained ambient air pollution data from the Center for Air, Climate and Energy Solutions (CACES) for particulate (PM_2.5_, PM_10_) and gaseous (O_3_, CO, SO_2_, and NO_2_) air pollutants. Annual‐average estimates were provided for each pollutant, except for O_3_ which was an average of the daily maximum 8‐h moving average from May through September for each year. Models were derived from publicly available US Environmental Protection Agency regulatory monitoring data and incorporate land use data (roads, elevation, urbanicity, etc.) and satellite‐derived estimates to predict concentrations in areas without ground‐level measurements. Each pollutant model was developed with the same unified framework using v1 empirical models as described in Kim et al. [16]. At the time of linkage, the CACES database (caces.us) included estimates for O_3_, SO_2_, NO_2_ for the years 1979–2015, PM_10_ for the years 1988–2015, CO for the years 1990–2015, and PM_2.5_ for the years 1999–2015 and were linked to the cohort based on residential address at the US census block group level. Address information in CPS‐II was first available in 1982 and then updated continuously from 1997 to 2015. Therefore, 1982 address data was used for years 1992–1996 until the beginning of updating in 1997. Of the participants still in the analysis in 1997, 78% were in the same census block group and 85% were in the same county as their 1982 address. Average pollutant levels for each year were assigned to participants based on their residential address during that year. In years when address changes occurred, the weighted average of each pollutant based on the number of months at each address was used as the annual value. PM_2.5_ values from 1991 to 1998 were back cast using the average ratio of PM_10_‐PM_2.5_ for each census block group from 1999 to 2003 [17]. The coarse fraction of particulate matter (PM_10‐2.5_) was calculated by subtracting PM_2.5_ from PM_10_. Data from 2015 were used for 2016 for all pollutants.

### 2.3 Outcomes

There were 28,008 incident first cancers (not including lung cancer) diagnosed in the analytic cohort between the start of follow‐up in 1992/1993 and the end of follow‐up on June 30, 2017. Primary identification of cases was through participant self‐report of cancer on surveys, followed by verification through medical record abstraction or linkage with state cancer registries (*n* = 23,223). Additional cases were participants who died between surveys that were identified through linkage with the National Death Index (NDI) with cancer listed as a cause of death (*n* = 4785). The majority of cases identified through NDI were subsequently verified through linkage with the state cancer registries (76%). Participants were only followed until their first cancer diagnosis. Cancer recurrences and diagnosis of additional cancers were not included in the analysis.

Cancer sub‐sites were identified using the International Classification of Disease for Oncology, Second and Third Edition for subjects with verified medical record or cancer registry data. The remaining participants with cancer information only from NDI (*n* = 1474) were categorized based on the International Classification of Diseases version 9 and 10. All sites analyzed separately had at least 50 cases except for cervical cancer, which was additionally included a priori, due to previously observed associations in the literature [18, 19]. Invasive breast cancer was additionally sub‐classified by estrogen receptor status (available in 82% of cases) and intrinsic subtype (available in 45% of cases).

### 2.4 Statistical Analysis

Ambient air pollutant concentrations were modeled as an average of all prior pollutant exposure starting at baseline and updated as a time‐varying exposure for each year of follow‐up. Person‐years of follow‐up were calculated from completion of the CPS‐II Nutrition Cohort questionnaire in 1992/1993 to date of: (1) diagnosis of any cancer; (2) death occurring between the last returned survey and next mailed survey; (3) date last returned a survey; or (4) end of follow‐up on June 30, 2017. Sex‐specific cancers were examined in men and women separately. Additional censoring criteria were included for female reproductive cancers. For uterine and cervical cancers, follow‐up was stopped if a woman reported a hysterectomy. For ovarian cancer, follow‐up was stopped if a woman reported having both ovaries removed.

Cox proportional hazards regression [20] was used to compute multivariable adjusted hazard ratios (HR) and 95% confidence intervals (CI) for the association between each ambient air pollutant and each cancer subtype. Days of follow‐up since enrollment were used as the model time scale. A risk set was formed at each event time of a cancer diagnosis, including all participants who were not censored, and a time‐varying average air pollutant exposure was constructed for each member of the risk set for exposures from 1991 (the year prior to the start of follow‐up) to the calendar year prior to the event year based on their residence over follow‐up time. The HRs were estimated using the difference between the 5th percentile and mean of each pollutant exposure during follow‐up.

All models were stratified on single‐year of age, and additionally adjusted for sex, race (white, black, other), education (high school or less/unknown, some college, college graduate), marital status (single, married, other), continuous body mass index (BMI), BMI squared, smoking status (never, quit 30+ years, quit 20–< 30 years, quit 10–< 20 years, quit < 10 years, current smoker, unknown), continuous cigarettes/day and years smoked with squared terms (to allow for a non‐linear relationship) in current smokers, smoking initiation before age 18 (no, yes), secondhand smoke exposure (hrs/week), ACS diet score based on adherence to recommended guidelines for fruit, vegetable, whole grain, red and processed meat, sugar‐sweetened beverage, and highly processed food intake (low, medium, high, unknown) [21], alcohol use (non‐drinker, < 1, 1–2, 2+, and unknown drinks/day), an occupational dirtiness index (0 = low exposure to 6 = high exposure) estimating the cumulative exposure to all occupational chemicals based on self‐reported main lifetime occupation [22], and any self‐reported regular exposure to occupational lung carcinogens (asbestos, chemicals/acids/solvents, coal/stone dusts, coal tar/pitch/asphalt, formaldehyde, or diesel engine exhaust) with a response of yes to any of the agents considered exposed (no, yes, unknown). Census tract level ecologic covariates from the US Census in 1990 and 2000, and the American Community Survey in 2010 were also included for median household income, percent college educated, percent of the population that is African American or other race, unemployment rate, and poverty rate and were updated throughout follow‐up to account for updated census information over time and residency changes. Smoking variables were updated every 2 years from 1997 to 2017. Additional covariates for reproductive cancers in women included oral contraceptive use (never, ever), hormone replacement therapy (never, ever use of estrogen only, ever use of combined estrogen and progesterone with no history of estrogen only, other types of hormone therapy/unknown), age at menarche (< 12, 12, 13, 14+, unknown), age at menopause (< 45, 45–50, 50–54, 55+, unknown), live births and age at 1st birth (none, 1–2 births at age < 25, 1–2 births at 25 or older, 3+ births starting before age 25, 3+ births starting at 25 or older, unknown). Screening variables were included in models and updated over time (recent–past 2 to 3 years, not recent, unknown) for specific cancers: breast included mammography, cervical included pap testing, prostate included prostate‐screening antigen (PSA) tests, and colorectal included colonoscopy/sigmoidoscopy.

Models are presented in all participants and among never smokers, and effect modification by region was assessed using multiplicative interaction terms. Alternative modeling using different covariates (models minimally adjusted for age and sex, and models without ecologic variables) and alternative exposure timing (rolling most recent 5‐year average) were also conducted. A sensitivity analysis stratified by whether participants moved was also conducted. A *p* value of < 0.05 was used to define statistical significance, and all *p* values are two‐sided. The proportional hazards assumption was evaluated by interactions with the log of fail‐time and using Schoenfeld residuals. No meaningful changes over time were observed. Statistical analysis was conducted using SAS (version 9.4) and R (version 4.4.0).

## 3 Results

**FIGURE 1:**
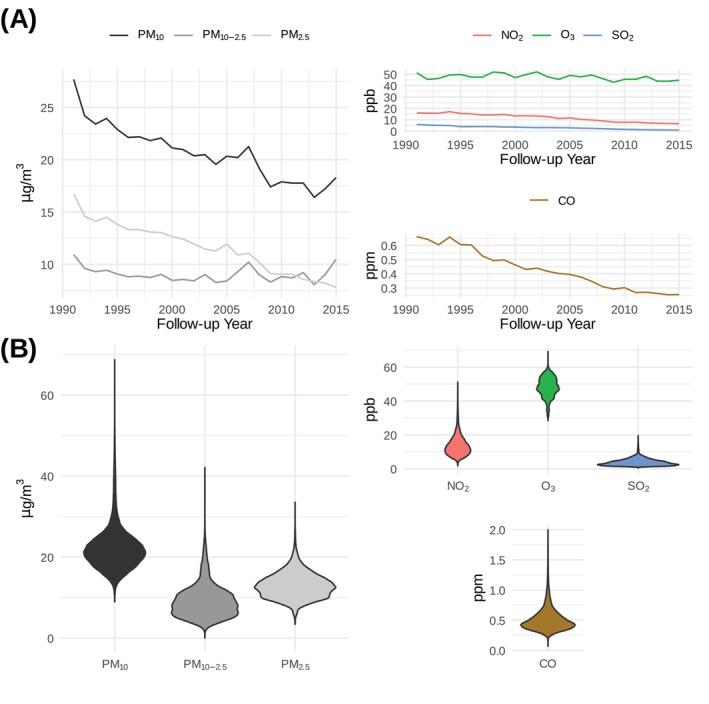
Distribution of ambient air pollutants in the CPS‐II Nutrition Cohort population. (A) Line plots of the mean pollutant values in cohort participants at each year of follow‐up from 1991 to 2015. (B) Violin plots of pollutant values for each participant averaged over their time in the study. The width of the violin plot represents the distribution of participants at that pollutant value. For both sets of plots, the particulate matter (PM_10_ = black, PM_10‐2.5_ = dark grey, PM_2.5_ = light grey) units are μg/m^3^, CO (brown) units are parts per million (ppm), and other gas (NO_2_ = red, O_3_ = green, SO_2_ = blue) units are parts per billion (ppb).

Distribution of the average pollutant exposures among CPS‐II participants during the follow‐up period is shown in Figure 1 and Table S1. The average annual pollutant values were generally higher in the early part of follow‐up and declined over time. Correlations between pollutants are provided in Table S2.

Participant characteristics at the start of follow‐up in 1992 are shown in Table 1 and Table S3. The participants were roughly equally divided between men and women. The average age of the population was 63 years (SD = 6.41) and primarily of white race with at least some college education. Over half of the population had a history of smoking cigarettes, but less than 10% were actively smoking at the start of follow‐up. Most of the women had a history of childbirth, and over half had used hormone replacement therapy. Over 80% of women reported a mammography in the last 2–3 years, and 65% of the men reported a PSA test. Participants were distributed throughout the United States; although a larger proportion resided in the Northeast (30%) and Midwest (32%).

**TABLE 1:** Selected participant characteristics at baseline in 1992 and median pollutant levels by characteristic.

Variable		Subjects	Pollutant levels at baseline with 1‐year lag (1991 Values)						
		*N* = 108,002	PM_2.5_ (μg/m^3^)	PM_10_ (μg/m^3^)	PM_10‐2.5_ (μg/m^3^)	NO_2_ (ppb)	O_3_ (ppb)	SO_2_ (ppb)	CO (ppm)
		*N* (%)	Median (25th–75th)	Median (25th–75th)	Median (25th–75th)	Median (25th–75th)	Median (25th–75th)	Median (25th–75th)	Median (25th–75th)
Age									
	< 60	33,280 (30.8)	16.7 (13.5–19.4)	26.0 (23.3–29.5)	9.9 (7.4–12.8)	14.5 (10.7–19.4)	51.9 (45.2–58.3)	6.1 (3.4–8.0)	0.60 (0.46–0.79)
	60‐ < 65	30,220 (28.0)	16.7 (13.5–19.4)	26.0 (23.4–29.5)	10.0 (7.5–12.8)	14.5 (10.7–19.3)	51.6 (44.6–57.7)	5.9 (3.3–7.9)	0.61 (0.47–0.79)
	65‐ < 70	27,182 (25.2)	16.8 (13.6–19.6)	26.3 (23.6–29.7)	10.0 (7.5–13.0)	14.6 (10.6–19.5)	51.5 (44.5–57.4)	5.9 (3.3–7.9)	0.61 (0.47–0.80)
	70+	17,320 (16.0)	16.9 (13.7–19.7)	26.4 (23.7–30.0)	10.1 (7.5–13.2)	14.4 (10.4–19.5)	51.2 (43.8–57.1)	5.6 (3.1–7.8)	0.62 (0.48–0.83)
Gender									
	Male	51,066 (47.3)	16.7 (13.5–19.5)	26.1 (23.4–29.5)	10.0 (7.4–12.9)	14.5 (10.6–19.4)	51.7 (44.7–57.8)	5.9 (3.3–7.9)	0.61 (0.47–0.80)
	Female	56,936 (52.7)	16.7 (13.5–19.5)	26.2 (23.5–29.6)	10.0 (7.5–12.9)	14.5 (10.6–19.4)	51.6 (44.5–57.7)	5.9 (3.3–7.9)	0.61 (0.47–0.80)
Race									
	White	104,856 (97.1)	16.7 (13.5–19.4)	26.1 (23.4–29.5)	10.0 (7.4–12.8)	14.4 (10.6–19.3)	51.7 (44.6–57.8)	5.9 (3.3–7.9)	0.61 (0.47–0.79)
	Non‐white	3146 (2.9)	18.4 (15.5–21.1)	28.5 (25.2–34.5)	11.8 (8.3–15.8)	17.9 (13.0–22.7)	50.5 (43.7–56.0)	4.8 (2.4–7.6)	0.74 (0.58–1.06)
Education									
	≤ High school	32,780 (30.4)	16.5 (13.2–19.2)	26.2 (23.4–29.5)	10.1 (7.8–12.8)	13.5 (9.7–18.6)	51.8 (45.4–58.0)	6.2 (3.9–8.1)	0.58 (0.44–0.75)
	Some college	31,265 (28.9)	16.7 (13.4–19.5)	26.3 (23.5–30.1)	10.4 (7.7–13.3)	14.6 (10.6–19.6)	51.1 (43.9–56.8)	5.4 (3.1–7.7)	0.62 (0.47–0.82)
	College graduate	43,957 (40.7)	17.0 (13.9–19.8)	26.1 (23.5–29.4)	9.6 (7.1–12.7)	15.2 (11.3–19.9)	51.9 (44.8–58.1)	6.0 (3.2–8.0)	0.63 (0.49–0.82)
Smoking status									
	Never	46,497 (43.1)	16.6 (13.4–19.5)	26.3 (23.6–29.9)	10.2 (7.6–13.2)	14.3 (10.5–19.3)	51.7 (44.6–57.7)	5.9 (3.3–7.8)	0.61 (0.47–0.80)
	Former	50,684 (46.9)	16.8 (13.7–19.5)	26.0 (23.3–29.4)	9.8 (7.3–12.7)	14.7 (10.7–19.6)	51.6 (44.5–57.8)	5.9 (3.3–7.8)	0.61 (0.47–0.80)
	Current	9439 (8.7)	16.9 (13.7–19.5)	26.3 (23.5–29.6)	10.1 (7.6–12.7)	14.4 (10.6–19.5)	51.7 (44.6–57.9)	6.1 (3.4–8.1)	0.60 (0.46–0.77)
Alcohol use									
	Non‐drinker	40,929 (37.9)	16.6 (13.4–19.4)	26.5 (23.8–30.1)	10.3 (7.8–13.3)	14.1 (10.2–19.1)	52.0 (45.1–57.5)	6.0 (3.5–7.8)	0.61 (0.46–0.79)
	Drinker	63,795 (59.1)	16.8 (13.4–19.5)	25.9 (23.3–29.3)	9.8 (7.3–12.6)	14.9 (11.0–19.7)	51.4 (44.4–57.9)	5.9 (3.2–8.0)	0.61 (0.47–0.80)
Body mass index									
	< 25	48,906 (45.3)	16.8 (13.7–19.6)	26.1 (23.5–29.6)	9.9 (7.3–12.9)	14.7 (10.8–19.6)	51.6 (44.4–57.6)	5.8 (3.3–7.9)	0.62 (0.48–0.81)
	25–< 30	42,087 (39.0)	16.7 (13.5–19.4)	26.1 (23.4–29.5)	10.0 (7.5–12.8)	14.4 (10.5–19.2)	51.7 (44.6–57.8)	6.0 (3.4–7.9)	0.60 (0.46–0.79)
	30+	15,463 (14.3)	16.6 (13.4–19.3)	26.3 (23.5–29.8)	10.2 (7.8–13.0)	14.3 (10.2–19.4)	51.7 (44.9–57.8)	6.1 (3.6–8.0)	0.60 (0.45–0.78)
Live in rural area at baseline									
	No	98,371 (91.1)	17.0 (13.9–19.7)	26.3 (23.7–29.9)	10.0 (7.4–12.9)	15.3 (11.5–19.9)	51.7 (44.8–58.0)	6.0 (3.3–8.0)	0.63 (0.49–0.83)
	Yes	9631 (8.9)	13.6 (11.3–16.8)	23.9 (21.5–27.3)	10.0 (8.0–12.4)	7.7 (6.6–9.5)	50.7 (43.2–55.1)	5.1 (3.6–7.0)	0.43 (0.33–0.52)
US region at baseline									
	Northeast	32,788 (30.4)	17.5 (15.2–19.5)	25.9 (22.3–28.8)	7.87 (6.42–9.76)	16.2 (12.0–21.4)	58.4 (52.4–61.2)	8.2 (7.4–9.2)	0.63 (0.47–0.81)
	South	19,800 (18.3)	18.7 (14.4–21.2)	26.0 (24.3–27.9)	7.75 (5.66–10.84)	12.9 (10.0–16.7)	53.0 (42.4–60.7)	5.4 (3.2–7.7)	0.57 (0.44–0.68)
	Midwest	34,112 (31.6)	14.1 (11.5–17.2)	24.8 (22.9–27.6)	11.0 (9.4–12.8)	12.1 (8.7–16.4)	49.2 (41.4–51.9)	4.7 (3.5–6.3)	0.52 (0.41–0.62)
	West	21,302 (19.7)	17.6 (14.5–22.9)	33.6 (27.0–42.8)	16.6 (11.7–22.3)	18.6 (13.0–24.1)	46.3 (42.7–54.2)	2.3 (1.7–3.9)	1.02 (0.81–1.22)

The strongest differences in pollutant exposures by individual participant characteristics were by race (Table 1). Non‐white racial groups were exposed to higher levels of most pollutants at baseline, with the strongest differences for PM_2.5_, PM_10_, and NO_2_. Urban areas had higher levels of most pollutants, and there were regional differences in pollutant exposure observed. Census tract characteristics associated with urbanicity were also associated with higher pollutant values, while poverty was associated with lower levels. Individual lifestyle factors were not strongly associated with different levels of pollutant exposure.

Associations between PM pollutants and incidence of cancer by type are shown in Table 2. There were no statistically significant associations with PM_2.5_ and any cancer site. There were positive but not statistically significant associations between uterine cancer and PM_10‐2.5_ (HR per 5.1 μg/m^3^ = 1.08; 95% CI 0.99–1.18) and HRs for cervical cancer were also elevated though CIs were wide with only 41 cervical cancer cases observed. Associations of PM_10‐2.5_ with gallbladder, pancreas, and laryngeal cancers were also positive at a higher magnitude but not statistically significant. PM_10_ and PM_10‐2.5_ were inversely associated with bladder cancer. There were no statistically significant associations with PM among never smokers (Table 2), though several PM_2.5_ estimates increased in a positive direction (liver, cervix, uterus, bladder, and kidney). In analyses by region (Table S4), there was a positive and statistically significant association of PM_2.5_ and liver cancer in the Midwest only (HR per 4.5 μg/m^3^ = 1.82; 95% CI 1.10–3.01; *p*‐interaction = 0.02).

**TABLE 2:** Association of particulate matter pollutants with subtypes of cancer in the CPS‐II Nutrition Cohort from 1992 to 2017.

Cancer site	All subjects					Never smokers				
	Person years	Cases	PM_2.5_ 4.5 μg/m^3^	PM_10_ 6.7 μg/m^3^	PM_10‐2.5_ 5.1 μg/m^3^	Person years	Cases	PM_2.5_ 4.5 μg/m^3^	PM_10_ 6.7 μg/m^3^	PM_10‐2.5_ 5.1 μg/m^3^
Non‐lung cancers	1,648,416	28,008	0.99 (0.97–1.01)	0.98 (0.97–1.00)	0.98 (0.97–1.00)	768,366	11,437	1.00 (0.97–1.03)	0.98 (0.96–1.01)	0.98 (0.96–1.00)
Head/Neck	1,648,416	314	1.01 (0.86–1.19)	0.89 (0.76–1.05)	0.86 (0.73–1.00)	768,366	97	1.01 (0.74–1.37)	0.89 (0.66–1.20)	0.87 (0.66–1.14)
Esophagus	1,648,416	264	1.01 (0.84–1.21)	0.99 (0.84–1.17)	0.99 (0.83–1.17)	768,366	56	0.78 (0.51–1.21)	0.78 (0.52–1.17)	0.89 (0.62–1.27)
Stomach	1,648,416	298	0.98 (0.83–1.16)	0.98 (0.84–1.14)	0.99 (0.85–1.15)	768,366	71	0.92 (0.65–1.31)	1.00 (0.74–1.34)	1.05 (0.79–1.40)
Colorectal	1,648,416	2636	1.02 (0.96–1.08)	1.03 (0.98–1.08)	1.03 (0.97–1.08)	768,366	1021	1.04 (0.95–1.14)	1.01 (0.93–1.10)	0.99 (0.91–1.07)
Liver	1,648,416	155	1.02 (0.80–1.30)	0.91 (0.73–1.14)	0.87 (0.70–1.09)	768,366	47	1.13 (0.73–1.74)	1.08 (0.76–1.54)	1.04 (0.72–1.49)
Gallbladder	1,648,416	64	1.03 (0.70–1.51)	1.19 (0.85–1.66)	1.21 (0.87–1.67)	768,366	30	1.02 (0.57–1.81)	1.42 (0.87–2.30)	1.45 (0.93–2.27)
Pancreas	1,648,416	799	0.90 (0.80–1.00)	0.99 (0.90–1.09)	1.07 (0.97–1.17)	768,366	354	0.97 (0.82–1.14)	0.99 (0.86–1.15)	1.01 (0.88–1.16)
Larynx	1,648,416	134	1.04 (0.81–1.35)	1.08 (0.86–1.36)	1.08 (0.85–1.36)	768,366	13	0.87 (0.38–2.01)	0.92 (0.43–1.95)	0.99 (0.48–2.02)
Melanoma	1,648,416	1994	1.02 (0.96–1.09)	0.96 (0.90–1.03)	0.94 (0.88–1.00)	768,366	867	1.05 (0.95–1.17)	0.98 (0.89–1.08)	0.94 (0.86–1.03)
Breast (Invasive)	941,561	4254	0.96 (0.92–1.01)	0.98 (0.94–1.02)	1.01 (0.97–1.05)	521,827	2202	0.93 (0.88–1.00)	0.95 (0.90–1.01)	0.98 (0.93–1.04)
Breast (In situ)	941,561	876	0.99 (0.90–1.09)	0.93 (0.85–1.02)	0.92 (0.84–1.00)	521,827	470	0.93 (0.82–1.07)	0.89 (0.79–1.01)	0.91 (0.81–1.03)
Cervix	594,765	41	1.09 (0.71–1.66)	1.23 (0.87–1.74)	1.27 (0.88–1.84)	323,944	20	1.41 (0.74–2.68)	1.36 (0.76–2.41)	1.14 (0.66–1.96)
Uterine	594,765	872	1.02 (0.92–1.12)	1.07 (0.98–1.17)	1.08 (0.99–1.18)	323,944	514	1.07 (0.95–1.22)	1.06 (0.94–1.18)	1.02 (0.91–1.14)
Ovarian	739,916	447	0.96 (0.84–1.11)	0.95 (0.83–1.07)	0.96 (0.84–1.09)	408,238	242	0.92 (0.76–1.11)	0.90 (0.76–1.08)	0.94 (0.79–1.11)
Prostate	706,855	7320	0.98 (0.95–1.01)	0.99 (0.96–1.02)	1.00 (0.97–1.03)	246,540	2654	0.99 (0.93–1.04)	0.98 (0.93–1.03)	0.99 (0.94–1.03)
Bladder	1,648,416	1607	0.96 (0.89–1.04)	0.91 (0.85–0.98)	0.92 (0.85–0.98)	768,366	364	1.09 (0.93–1.27)	1.08 (0.94–1.24)	1.04 (0.91–1.19)
Kidney	1,648,416	564	1.06 (0.93–1.21)	0.96 (0.85–1.08)	0.91 (0.81–1.02)	768,366	223	1.15 (0.94–1.40)	1.04 (0.87–1.25)	0.96 (0.81–1.14)
Brain	1,648,416	310	0.93 (0.79–1.10)	0.99 (0.85–1.16)	1.04 (0.90–1.21)	768,366	140	0.92 (0.71–1.18)	1.08 (0.86–1.35)	1.14 (0.93–1.40)
Thyroid	1,648,416	219	0.96 (0.78–1.18)	0.97 (0.80–1.17)	0.99 (0.82–1.18)	768,366	111	1.09 (0.82–1.45)	1.04 (0.81–1.34)	0.99 (0.78–1.27)
Hematologic	1,648,416	3128	0.98 (0.93–1.04)	0.97 (0.92–1.02)	0.97 (0.92–1.02)	768,366	1304	0.99 (0.91–1.07)	0.98 (0.91–1.06)	0.99 (0.92–1.06)

*Note:* Stratified on age in 1992, adjusted for gender, race, education, marital status, BMI, smoking status, years smoked, cigarettes/day, years since quit, started smoking < 18, years passive smoking, ACS diet score, alcohol, occupational dirtiness, occupational exposure and census tract data (median household income, % college educated, % African American, % Other non‐white race, unemployment rate, poverty rate). Female cancers additionally adjusted for: oral contraceptives, HRT Use, age at menarche, age at menopause, age at 1st birth and number of live births. Specific cancer sites with additional adjustment: breast cancer (mammography), cervical cancer (pap test), prostate cancer (PSA test), colorectal cancer (colonoscopy/sigmoidoscopy).

There were few statistically significant associations with gaseous pollutants and cancer subtypes (Table 3). Ozone was positively associated with melanoma of the skin in all participants (O_3_ HR per 9.9 ppb = 1.08; 95% CI 1.00–1.17). Among never smokers, there were some elevated HRs for NO_2_ and melanoma of the skin (HR per 7.2 ppb = 1.08; 95% CI 0.98–1.18) and kidney cancer (HR per 7.2 ppb = 1.19; 95% CI 1.00–1.42) as well as CO and kidney cancer (HR per 0.21 ppm = 1.17; 95% CI 1.00–1.37). SO_2_ was associated with a higher risk of colorectal cancer in never smokers as well (HR per 2.3 ppb = 1.08; 95% CI 1.01–1.16).

**TABLE 3:** Association of gaseous pollutants with subtypes of cancer in the CPS‐II Nutrition Cohort from 1992 to 2017.

Cancer site	All subjects						Never smokers					
	Person years	Cases	NO_2_ 7.2 ppb	Ozone 9.9 ppb	SO_2_ 2.3 ppb	CO 0.21 ppm	Person years	Cases	NO_2_ 7.2 ppb	Ozone 9.9 ppb	SO_2_ 2.3 ppb	CO 0.21 ppm
Non‐lung cancers	1,648,416	28,008	1.00 (0.98–1.01)	0.99 (0.97–1.01)	1.00 (0.98–1.01)	1.01 (0.99–1.02)	768,366	11,437	1.00 (0.98–1.03)	0.99 (0.96–1.03)	1.00 (0.98–1.02)	1.00 (0.98–1.02)
Head/Neck	1,648,416	314	0.87 (0.74–1.02)	1.01 (0.84–1.22)	0.89 (0.78–1.02)	0.92 (0.80–1.06)	768,366	97	0.81 (0.60–1.10)	1.02 (0.72–1.43)	0.81 (0.62–1.06)	0.94 (0.72–1.22)
Esophagus	1,648,416	264	0.98 (0.83–1.16)	0.95 (0.77–1.17)	1.03 (0.89–1.18)	1.03 (0.88–1.19)	768,366	56	0.72 (0.48–1.09)	0.96 (0.61–1.50)	0.95 (0.68–1.33)	0.84 (0.58–1.22)
Stomach	1,648,416	298	1.00 (0.85–1.16)	0.86 (0.71–1.05)	0.89 (0.77–1.02)	1.00 (0.87–1.14)	768,366	71	1.00 (0.73–1.36)	0.70 (0.48–1.04)	0.78 (0.56–1.07)	1.16 (0.90–1.50)
Colorectal	1,648,416	2636	1.02 (0.96–1.07)	1.01 (0.94–1.07)	1.04 (0.99–1.08)	1.03 (0.99–1.08)	768,366	1021	1.02 (0.94–1.11)	1.07 (0.96–1.19)	1.08 (1.01–1.16)	1.02 (0.95–1.10)
Liver	1,648,416	155	0.98 (0.78–1.22)	0.87 (0.66–1.13)	1.08 (0.90–1.31)	0.84 (0.68–1.04)	768,366	47	0.99 (0.68–1.45)	0.97 (0.59–1.61)	0.92 (0.63–1.35)	1.03 (0.74–1.45)
Gallbladder	1,648,416	64	1.08 (0.75–1.54)	0.90 (0.60–1.35)	1.01 (0.76–1.36)	1.03 (0.75–1.43)	768,366	30	1.26 (0.74–2.12)	0.92 (0.51–1.69)	0.95 (0.61–1.46)	0.89 (0.53–1.49)
Pancreas	1,648,416	799	1.03 (0.93–1.14)	0.90 (0.80–1.01)	0.96 (0.88–1.05)	1.06 (0.97–1.16)	768,366	354	1.10 (0.95–1.28)	0.97 (0.81–1.16)	1.01 (0.89–1.16)	1.09 (0.96–1.25)
Larynx	1,648,416	134	0.99 (0.79–1.26)	1.04 (0.78–1.38)	1.12 (0.93–1.34)	1.09 (0.89–1.33)	768,366	13	0.72 (0.32–1.63)	0.91 (0.37–2.20)	1.21 (0.68–2.13)	0.85 (0.43–1.65)
Melanoma	1,648,416	1994	1.02 (0.96–1.09)	1.08 (1.00–1.17)	1.01 (0.96–1.07)	1.04 (0.98–1.10)	768,366	867	1.08 (0.98–1.18)	1.11 (0.99–1.25)	1.03 (0.95–1.11)	1.04 (0.96–1.13)
Breast (Invasive)	941,561	4254	0.97 (0.93–1.02)	0.96 (0.91–1.01)	0.97 (0.93–1.00)	1.00 (0.96–1.04)	521,827	2202	0.97 (0.91–1.03)	0.92 (0.86–0.98)	0.99 (0.94–1.04)	0.99 (0.94–1.04)
Breast (In situ)	941,561	876	0.95 (0.87–1.05)	1.02 (0.91–1.14)	0.98 (0.91–1.06)	0.97 (0.89–1.05)	521,827	470	0.90 (0.80–1.03)	0.99 (0.86–1.15)	0.97 (0.87–1.08)	0.95 (0.85–1.06)
Cervix	594,765	41	0.75 (0.49–1.15)	0.79 (0.48–1.30)	0.80 (0.56–1.12)	0.91 (0.65–1.28)	323,944	20	0.49 (0.23–1.05)	1.07 (0.52–2.22)	0.84 (0.51–1.39)	0.69 (0.39–1.24)
Uterine	594,765	872	1.04 (0.95–1.13)	1.01 (0.91–1.13)	0.97 (0.90–1.05)	1.06 (0.98–1.14)	323,944	514	1.07 (0.95–1.20)	1.00 (0.86–1.15)	1.01 (0.92–1.11)	1.04 (0.94–1.15)
Ovarian	739,916	447	0.95 (0.84–1.08)	0.95 (0.81–1.10)	0.90 (0.81–1.01)	0.95 (0.85–1.07)	408,238	242	0.89 (0.74–1.07)	0.97 (0.79–1.19)	0.88 (0.76–1.03)	0.87 (0.74–1.02)
Prostate	706,855	7320	0.99 (0.95–1.02)	1.01 (0.98–1.05)	1.00 (0.98–1.03)	0.99 (0.96–1.01)	246,540	2654	0.96 (0.91–1.01)	1.01 (0.95–1.07)	0.99 (0.95–1.04)	0.97 (0.93–1.01)
Bladder	1,648,416	1607	0.99 (0.92–1.06)	0.92 (0.85–1.00)	1.03 (0.97–1.09)	1.00 (0.93–1.06)	768,366	364	1.09 (0.94–1.25)	0.97 (0.81–1.16)	0.99 (0.87–1.12)	1.00 (0.88–1.15)
Kidney	1,648,416	564	1.06 (0.94–1.19)	1.04 (0.91–1.20)	1.08 (0.98–1.19)	1.08 (0.97–1.20)	768,366	223	1.19 (1.00–1.42)	1.01 (0.80–1.26)	1.07 (0.92–1.25)	1.17 (1.00–1.37)
Brain	1,648,416	310	1.04 (0.89–1.22)	1.09 (0.90–1.31)	1.01 (0.89–1.15)	1.06 (0.93–1.21)	768,366	140	1.05 (0.83–1.32)	1.19 (0.90–1.57)	1.09 (0.91–1.31)	1.04 (0.85–1.27)
Thyroid	1,648,416	219	0.97 (0.80–1.17)	1.09 (0.87–1.38)	0.96 (0.82–1.13)	0.97 (0.82–1.15)	768,366	111	1.05 (0.81–1.36)	1.18 (0.85–1.64)	1.04 (0.83–1.30)	1.03 (0.82–1.29)
Hematologic	1,648,416	3128	1.00 (0.95–1.06)	0.98 (0.92–1.04)	0.97 (0.93–1.01)	1.03 (0.98–1.08)	768,366	1304	1.02 (0.94–1.10)	1.00 (0.91–1.09)	0.99 (0.93–1.06)	1.04 (0.97–1.11)

*Note:* Stratified on age in 1992, adjusted for gender, race, education, marital status, BMI, smoking status, years smoked, cigarettes/day, years since quit, started smoking < 18, years passive smoking, ACS diet score, alcohol, occupational dirtiness, occupational exposure and census tract data (median household income, % college educated, % African American, % Other non‐white race, unemployment rate, poverty rate). Female cancers additionally adjusted for: oral contraceptives, HRT Use, age at menarche, age at menopause, age at 1st birth and number of live births. Specific cancer sites with additional adjustment: breast cancer (mammography), cervical cancer (pap test), prostate cancer (PSA test), colorectal cancer (colonoscopy/sigmoidoscopy).

There were few associations of air pollution with breast cancer hormone receptor status (Table 4). The strongest positive association was with PM_10‐2.5_ and estrogen receptor negative (ER−) disease (HR per 5.1 μg/m^3^ = 1.16; 95% CI 1.03–1.30), although PM_2.5_ was inversely associated with ER‐. CO was positively associated with ER+ breast cancer (HR per 0.21 ppm = 1.04; 95% CI 0.99–1.08) although the association was not statistically significant. SO_2_ was inversely associated with both ER+ and ER− disease.

**TABLE 4:** Association of air pollutants with invasive breast cancer subtypes in the CPS‐II Nutrition Cohort from 1992 to 2017.

Cancer site	Person years	Cases	PM_2.5_ 4.5 μg/m3	PM_10_ 6.7 μg/m^3^	PM_10‐2.5_ 5.1 μg/m^3^	NO_2_ 7.2 ppb	Ozone 9.9 ppb	SO_2_ 2.3 ppb	CO 0.21 ppm
ER status									
Estrogen receptor positive (ER+)	941,561	3032	0.98 (0.92–1.03)	1.00 (0.96–1.05)	1.02 (0.97–1.07)	0.98 (0.93–1.03)	0.95 (0.89–1.01)	0.94 (0.89–0.98)	1.04 (0.99–1.08)
Estrogen receptor negative (ER−)	941,561	477	0.85 (0.74–0.97)	1.03 (0.91–1.16)	1.16 (1.03–1.30)	0.94 (0.82–1.07)	0.90 (0.77–1.04)	0.85 (0.76–0.95)	1.06 (0.95–1.18)
Intrinsic subtype									
Luminal A	941,561	1518	0.96 (0.88–1.04)	0.97 (0.91–1.04)	0.99 (0.93–1.07)	0.96 (0.89–1.03)	0.92 (0.85–1.01)	0.95 (0.89–1.02)	1.02 (0.95–1.09)
Luminal B	941,561	186	1.00 (0.80–1.26)	0.95 (0.76–1.19)	0.94 (0.76–1.16)	0.98 (0.78–1.22)	1.02 (0.80–1.30)	1.00 (0.83–1.19)	0.99 (0.81–1.21)
HER‐2 enriched	941,561	55	0.60 (0.38–0.96)	0.69 (0.42–1.11)	0.96 (0.64–1.44)	0.83 (0.54–1.28)	0.81 (0.51–1.27)	0.77 (0.52–1.13)	0.86 (0.57–1.30)
Triple negative	941,561	156	0.94 (0.73–1.20)	1.04 (0.83–1.31)	1.10 (0.88–1.36)	0.99 (0.77–1.26)	1.06 (0.80–1.39)	1.05 (0.86–1.28)	1.09 (0.87–1.35)

*Note:* Stratified on age in 1992, adjusted for gender, race, education, marital status, BMI, smoking status, years smoked, cigarettes/day, years since quit, started smoking < 18, years passive smoking, ACS diet score, alcohol, occupational dirtiness, occupational exposure, oral contraceptives, HRT Use, age at menarche, age at menopause, age at 1st birth and number of live births, mammography and census tract data (median household income, % college educated, % African American, % Other non‐white race, unemployment rate, poverty rate).

Sensitivity analyses did not substantively change the results. Alternate model adjustment including only age and sex, as well as a fully adjusted model that did not include area‐level covariates, were generally in a similar direction to the main models although some associations were stronger without ecologic adjustments (Table S5). An alternative exposure using a 5‐year moving pollutant average to examine more recent exposure during follow‐up also did not materially change the results (Table S6). A sensitivity analysis starting follow‐up when address updating began in 1997 also showed similar patterns of association (Table S7).

## 4 Discussion

Overall, there were few positive associations with air pollutants and incident cancer subtypes in the present analysis. There were no positive associations observed with PM_2.5_ at any non‐lung cancer site, though associations were stronger for several sites among never smokers. PM_10_ and PM_10‐2.5_ were positively associated with uterine cancer and cervical cancer overall, although associations were generally attenuated in never smokers. Among breast cancer subtypes, there was an association with PM_10‐2.5_ and ER− disease. There were also few associations observed with gaseous pollutants. Ozone and NO_2_ were positively associated with melanoma of the skin. In never smokers, NO_2_ and CO were positively associated with kidney cancer and SO_2_ with colorectal cancer. In general, the magnitude of pollutant associations increased in non‐smokers, suggesting the potential for residual confounding by smoking status. These findings suggest pollutant associations beyond lung cancer may be low in magnitude and limited to specific cancer types and populations.

A primary hypothesis of this study was that pollutants could play a different role in incident cancer risk than death from cancer. Previous research in the larger CPS‐II mortality cohort of over 600,000 participants that included over 40,000 cancer deaths identified significant associations with PM_2.5_ and bladder (*n* = 1324) and kidney (*n* = 927) cancer death and of NO_2_ and colorectal (*n* = 6475) cancer death [13]. We did not observe the same statistically significant associations in the current study of cancer incidence here (*n* = 1607 incident bladder, 564 kidney, and 2636 colorectal), though some weak positive associations were observed for particulate or gaseous pollutants in some cases. Fewer cases in this study and low magnitude associations may indicate a lack of statistical power to repeat those findings. Exposure data was from a different source in this study which covered a longer time‐period (particularly for O_3_ and NO_2_), was more aligned with follow‐up, and accounted for residential mobility. However, the previous and current exposures are highly correlated, suggesting exposure measures are not the source of differences in the findings. It is possible that aging of the cohort may play a role in the difference between the studies, as well as declines in air pollution concentrations and changes in composition over time. The mortality cohort was 52 years old on average at the start of follow‐up while the incidence population was aged 63 years on average. The higher background cancer rate in the older population could reduce the observable effects of ambient air pollution in the study population. Additionally, this study has more cancers from screening such as breast, prostate, and colorectal cancer that are less fatal, possibly resulting in differences with the mortality study. This is supported by associations of air pollutants with lung cancer, which is highly fatal, that were similar for both the mortality and incidence analyses previously published in this cohort [4, 14]. However, different findings for incidence and mortality may also suggest that air pollution is differentially affecting cancer initiation and cancer progression at these sites. Research on lung cancer has found PM_2.5_ is involved in promotion of pre‐existing disease rather than the initial mutation which could more strongly influence mortality [23], although this could vary by cancer site.

### 4.1 PM and Cancer

There have been several studies on colorectal cancer that generally align with what was observed in this study. The previous CPS‐II study identified an association with PM_2.5_ and colorectal cancer mortality (HR per 4.4 μg/m^3^ = 1.04, 95% CI 1.00–1.08) [13], which was of similar magnitude to the current study suggesting differences in significance may only be due to statistical power. A recent meta‐analysis of PM_2.5_ and colorectal cancer reported a larger relative risk of 1.42 (95% CI 1.12–1.79) per 10 μg/m^3^ increase in PM_2.5_, although there was high heterogeneity between studies [24]. Two [18, 25] of the seven studies in the meta‐analysis had substantially higher RRs than the others suggesting possible methodological differences, while the remaining studies were of similar magnitude to CPS‐II. Studies of PM_10_ and colorectal cancer incidence in the UK Biobank [24] and the German state of Saxony [26] found no association, nor did studies of PM_10_ and colorectal cancer mortality [27, 28]. We did not find an association with PM_10_ in our main results, but in a model without ecologic adjustment we found stronger associations with PM_10‐2.5_. Particulates can consist of a variety of toxic compounds including polycyclic aromatic hydrocarbons, metals, and other carcinogens that interact with the gastrointestinal system by ingestion. These particles can impact cancer growth through multiple mechanisms including inflammation (both locally and systemically), generation of mitochondrial reactive oxygen species, the addition of DNA adducts, or by being directly mutagenic [1, 7, 29]. There is also growing evidence that particulate air pollution is associated with poor gut microbiome diversity, which may play a role in cancer development [30].

Research on PM and gynecologic cancers has focused on PM_2.5_, and the few cervical cancer studies have generally indicated higher risks. In the CPS‐II mortality cohort [13] an elevated HR for cervical cancer death was observed (PM_2.5_ HR per 4.4 μg/m^3^ = 1.34; 95% CI 0.98–1.83). A prospective US study and a Brazilian ecologic study of PM_2.5_ and cervical cancer mortality observed strongly increased HRs for more highly exposed individuals [18, 19], as did research using US cancer registry data [31]. However, findings in this study were limited by the small numbers of incident cervical cancer cases (*n* = 41), although the results in never smokers were of similar high magnitude. Studies of uterine cancer have been less consistent although two indicated increased risk [19, 32]. PM exposure has been related to immune function [1, 7]. For cervical cancer, subclinical immune dysfunction because of air pollution could be impacting the ability to clear HPV infection, which is the primary risk factor for cervical cancer. Cigarette smoking is an established risk factor for cervical cancer and suppressed immune function is thought to be a mechanism of action [33, 34].

A 2021 meta‐analysis of outdoor air pollution and breast cancer found no association with PM_2.5_ [8] which is consistent with the main results in this cohort. However, it has also been suggested that associations with PM_2.5_ and breast cancer are dependent on the specific timing of exposure, specific subtypes, or specific geographic regions [8, 35]. A 2024 study of historic PM_2.5_ exposure found stronger associations with breast cancer suggesting early life exposure may be important [35], but we were unable to address this in the current study. A pooled study of US cohorts from 2025 found PM_2.5_ was associated with an increased risk of ER‐ breast cancer, whereas our study had an inverse association [36]. Hormonal status may be causing the contradictory findings. The pooled analysis had the strongest associations with ER‐ breast cancer in premenopausal women whereas our population is post‐menopausal [36]. Our positive association with PM_10‐2.5_ and ER‐ disease has not been observed previously. Biogenic coarse particulates (ex. pollen) are associated with immune and inflammatory dysfunction and possibly more relevant for the older CPS‐II population [1]. Although these opposing findings for PM_2.5_ and PM_10‐2.5_ with ER‐ breast cancer could also be due to chance. There were also no overall associations between gaseous pollutants and breast cancer in this study. However, meta‐analyses of other previous research on breast cancer indicated small associations with NO_2_ of 1%–3% increase in risk per 10 μg/m^3^ [8, 10, 36].

Liver cancer incidence has been related to PM_2.5_ in several previous studies [37] but was only present in the Midwest area in this study. PM is made up of different components regionally and it is possible that unique regional industries such as steel or automobile manufacturing are playing a role [38]. Although there was an overall association of PM_2.5_ and bladder cancer in the larger CPS‐II mortality study [13], there were no clear findings here. Survival rates are high in bladder cancer suggesting that PM_2.5_ may be impacting disease progression only observed in mortality studies [39]. The literature is inconclusive regarding an association between PM_2.5_ and bladder cancer [12].

### 4.2 Gaseous Pollutants and Cancer

Outdoor air pollution associations with both incident and fatal kidney cancer were reviewed in 2020 [12] with the conclusion that there was suggestive but heterogeneous evidence for an association with NO_2_/NO_x_. In the larger CPS‐II mortality cohort, there was no association with NO_2_ and kidney cancer with regard to all subjects. However, there were some elevated HRs for gaseous pollutant exposure in this study, including a 19% increase in risk of kidney cancer per 7.2 ppb of NO_2_ in never smokers (HR = 1.19; 95% CI 1.00–1.42). This suggests a role in incidence not observed in fatal disease, given the moderately high survival rate in kidney cancer [39]. In other recent studies, hospitalizations due to kidney cancer in Italy were associated with increased NO_2_ (HR per 10 μg/m^3^ = 1.20; 95% CI 1.07–1.33) [40]. The ELAPSE cohort found no association with NO_2_, although results were elevated in areas with levels below 20 μg/m^3^ [41]. NO_2_ has been associated with chronic kidney disease, a risk factor for kidney cancer, although the exact mechanism for this relationship is unknown [42]. NO_2_ is also typically accompanied by PAHs in vehicle emissions. PAHs are stored in the kidneys and are bio‐activated by renal metabolic pathways, which can lead to DNA adducts and possibly carcinogenesis [43].

We also observed associations with several gaseous pollutants and melanoma. There have been few other studies on air pollution and melanoma risk, and results are mixed. There was no association in the CPS‐II mortality cohort, nor a study of prevalent disease in the United Kingdom Biobank [13, 44], although the different outcome and study designs may explain the difference between the results from these studies and the present findings, as melanoma has a high survival rate [45]. The skin is directly exposed to outdoor air pollutants which may affect the skin in multiple ways: interacting with compounds in the skin to form reactive oxygen species, interfering with aryl hydrocarbon receptor signaling, and impacting vitamin D production [46]. O_3_ and CO were most strongly associated with melanoma and are highly reactive gases which could plausibly act through these mechanisms. However, these findings were unexpected and may also be due to chance.

### 4.3 Strengths and Limitations

This study has several strengths. It is a large prospective cohort that comprehensively examined many cancer sites with several pollutants with large numbers of cancer cases observed for common cancers. The ability to examine pollutant associations in never smoking participants is a major strength. In general, associations were strengthened in never smokers suggesting that lower magnitude pollutant associations may be more observable in that population. There were methodological strengths related to the timing and updating of exposures and outcomes. Concerns about the prior literature include one‐time measures of pollutant exposures, the misalignment of the exposure data with the timing of the outcome, or an inability to account for the changing levels of the exposure over time. In this study, we used a cumulative average over time to account for changing exposure levels both due to temporality and residential address changes. The population is also widely distributed throughout the US, enhancing the exposure contrast over a long period of time. The study used incident cases as well as updated screening information which allowed examination of associations with less fatal cancers that may not be picked up in mortality analyses.

Nonetheless, this study has several limitations. Specific constituents of PM were not examined that may be more relevant for some cancers and could explain differences in PM_2.5_ and PM_10‐2.5_ associations. PM_2.5_ components have been more strongly associated with traffic emissions, while PM_10‐2.5_ is more strongly associated with crustal factors and abrasive vehicular emissions, although constituents of both vary by urbanicity [47, 48]. There are also changes in the composition of PM over time, and additional research on this topic is needed [49]. The population in this study was on average 63 years old at the start of follow‐up, and it is possible we are missing associations in younger populations or in specific time‐periods such as around puberty or menopause. There are few non‐White participants in this study, who tend to have higher pollutant exposures, which limits the generalizability of these findings. This study included many comparisons indicating that some of the observed results may be caused by chance. Our strongest a priori hypotheses were with PM_2.5_ and indicators of traffic‐related pollutants (NO_2_ and CO) with smoking‐related cancers. This supports our findings with colorectal and kidney cancer, while associations with ozone (melanoma) and coarse particulates (ER− breast and cervical cancer) were more exploratory and should be interpreted cautiously. Cancers are heterogeneous, and it is possible more detailed examinations of subtypes would identify different relationships [50]. Although analyses examined air pollution exposures during the entire follow‐up time, as well as the most recent 5‐year period, further work to explore potential latency effects for different cancer sites may also be useful. Several modeling assumptions were made related to back‐casting PM_2.5_, applying estimates from 2015 to 2016, using estimates of ozone only in the summer months, and using 1982 address through 1997 which could result in exposure misclassification. Census block groups capture similar sized population groups but may vary in spatial size with rural areas covering larger spaces. This could result in possible underestimation of exposure that is not present in urban areas. There were also small numbers of incident cases for several of the examined sites which reduced the statistical power.

In conclusion, this comprehensive study of particulate and gaseous air pollutants and incident cancer found few pollutant associations with specific cancer sites. However, a few statistically significant low‐magnitude associations were observed at some sites, and several strengthened in never smokers. Future research should focus on large‐scale and pooled studies, particularly in never smokers and for rare cancers, as well as examining impacts on other aspects of cancer such as survival after a diagnosis.

## Author Contributions

**W. Ryan Diver:** conceptualization, methodology, data curation, formal analysis, visualization, writing – original draft. **Lauren R. Teras:** data curation, writing – review and editing. **Emily L. Deubler:** data curation, writing – review and editing, formal analysis. **Alpa V. Patel:** data curation, writing – review and editing. **Michelle C. Turner:** conceptualization, methodology, writing – review and editing, supervision.

## Funding

The American Cancer Society funds the creation, maintenance, and updating of the Cancer Prevention Study‐II cohort. M.C.T. is funded by a Ramón y Cajal fellowship (RYC‐2017‐01892) from the Spanish Ministry of Science, Innovation and Universities and co‐funded by the European Social Fund. ISGlobal acknowledges support from the grant CEX2023‐0001290‐S funded by MCIN/AEI/10.13039/501100011033, and support from the Generalitat de Catalunya through the CERCA Program.

## Ethics Statement

The study protocol was approved by the institutional review boards of Emory University (IRB00045780), and those of participating cancer registries as required. Written informed consent was received from participants to obtain medical records. At the time of each mailed survey, participants were informed that their identifying information would be used to link with cancer registries and death indexes.

## Conflicts of Interest

The authors declare no conflicts of interest.

## Supporting information


**Figure S1:** Cohort exclusions and outcome verification.
**Table S1:** Distribution of average air pollutant values for the CPS‐II Nutrition Cohort during follow‐up.
**Table S2:** Pearson correlation coefficients between pollutants (average exposure from 1992 to 2015) among CPS‐II participants.
**Table S3:** Screening, reproductive, and census tract characteristics at baseline and median pollutant values by characteristics.
**Table S4:** Association of PM2.5 with subtypes of cancer by US Region in the CPS‐II Nutrition Cohort from 1992 to 2017.
**Table S5:** Alternative covariate models for the association of air pollutants with subtypes of cancer in the CPS‐II Nutrition Cohort from 1992 to 2017.
**Table S6:** Alternative exposure timing with 5‐year moving averages for the association with air pollutants with subtypes of cancer in the CPS‐II Nutrition Cohort from 1992 to 2017.
**Table S7:** Limiting follow‐up to the period with updated address history (1997–2017) for the association of air pollutants with subtypes of cancer in the CPS‐II Nutrition Cohort.

## Data Availability

Data are available from the American Cancer Society by following the ACS Data Access Procedures (https://www.cancer.org/research/population‐science/research‐collaboration.html) for researchers who meet the criteria for access to confidential data. Please email cohort.data@cancer.org to inquire about access. Further information is available from the corresponding author upon request.
