# Supplementary material for: Associations of Outdoor Air Pollution With Incidence of Cancers Other Than Lung Cancer in a Large US Prospective Cohort
Source: Int J Cancer. 2026 May 3;159(6):1440–54. doi: 10.1002/ijc.70530 (PMC13397290; doi:10.1002/ijc.70530)
Supplement: Supplementary file 1 — Figure S1: Cohort exclusions and outcome verification. Table S1: Distribution of average air pollutant values for the CPS‐II Nutrition Cohort during follow‐up. Table S2: Pearson correlation coefficients between pollutants (average exposure from 1992 to 2015) among CPS‐II participants. Table S3: Screening, reproductive, and census tract characteristics at baseline and median pollutant values by characteristics. Table S4: Association of PM2.5 with subtypes of cancer by US Region in the CPS‐II Nutrition Cohort from 1992 to 2017. Table S5: Alternative covariate models for the association of air pollutants with subtypes of cancer in the CPS‐II Nutrition Cohort from 1992 to 2017. Table S6: Alternative exposure timing with 5‐year moving averages for the association with air pollutants with subtypes of cancer in the CPS‐II Nutrition Cohort from 1992 to 2017. Table S7: Limiting follow‐up to the period with updated address history (1997–2017) for the association of air pollutants with subtypes of cancer in the CPS‐II Nutrition Cohort. [file IJC-159-1440-s001.pdf]

Supplementary Materials

Associations of outdoor air pollution with incidence of cancers other than lung cancer in a large US prospective cohort

Authors: W. Ryan Diver, Lauren R. Teras, Emily L. Deubler, Alpa V. Patel, Michelle C. Turner

Table of Contents

Supplemental Figure 1. Cohort exclusions and outcome verification

Supplemental Table 1. Distribution of average air pollutant values for the CPS-II Nutrition Cohort during follow-up

Supplemental Table 2. Pearson correlation coefficients between pollutants (average exposure from 1992-2015) among CPS-II participants.

Supplemental Table 3. Screening, reproductive, and census tract characteristics at baseline and median pollutant values by characteristics.

Supplemental Table 4. Association of PM2.5 with subtypes of cancer by US Region in the CPS-II Nutrition Cohort from 1992-2017

Supplemental Table 5. Alternative covariate models for the association of air pollutants with subtypes of cancer in the CPS-II Nutrition Cohort from 1992-2017.

Supplemental Table 6. Alternative exposure timing with 5-year moving averages for the association with air pollutants with subtypes of cancer in the CPS-II Nutrition Cohort from 1992-2017.

Supplemental Table 7. Limiting follow-up to the period with updated address history (1997-2017) for the association of air pollutants with subtypes of cancer in the CPS-II Nutrition Cohort.

Supplemental Figure 1. Cohort exclusions and outcome verification

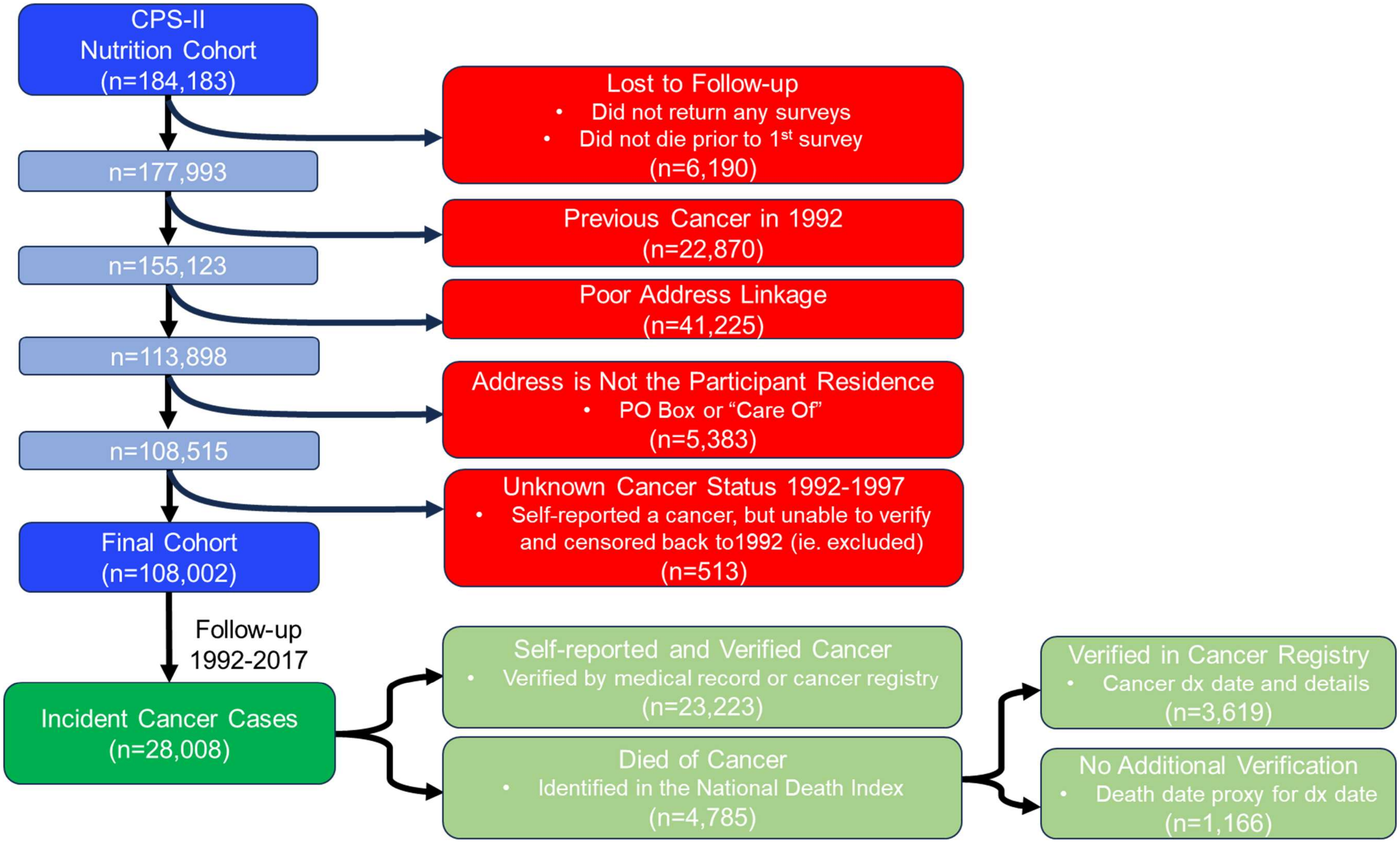

Supplemental Table 1. Distribution of average air pollutant values for the CPS-II Nutrition Cohort during follow-up

Variable	Mean	Min	5th	25th	50th	75th	95th	Max	5th-Mean
$PM_{2.5}$ (μg/m^3)	13.0	3.4	8.5	10.7	12.7	14.9	18.4	33.6	4.5
PM_{10} (μg/m^3)	22.1	8.9	15.5	18.8	21.4	24.3	31.7	68.7	6.7
$PM_{10-2.5}$ (μg/m^3)	9.2	0.0	4.0	6.2	8.6	11.2	17.4	42.1	5.1
NO_2 (ppb)	13.6	1.7	6.5	9.5	12.6	16.5	24.4	51.1	7.2
O_3 (ppb)	48.2	28.4	38.3	44.6	48.4	52.5	56.7	69.1	9.9
SO_2 (ppb)	3.8	0.5	1.5	2.3	3.4	4.8	7.4	19.5	2.3
CO (ppm)	0.51	0.07	0.30	0.39	0.47	0.59	0.89	2.00	0.21

*Values for pollutants are an average for each participant from baseline to time of event or censoring.

Supplemental Table 2. Pearson correlation coefficients between pollutants (average exposure from 1992-2015) among CPS-II participants.

	$PM_{2.5}$	PM_{10}	$PM_{10-2.5}$	NO_2	O_3	SO_2	CO
$PM_{2.5}$	1.00						
PM_{10}	0.65	1.00					
$PM_{10-2.5}$	0.08	0.81	1.00				
NO_2	0.63	0.66	0.37	1.00			
O_3	0.50	0.18	-0.15	0.18	1.00		
SO_2	0.47	0.05	-0.30	0.27	0.44	1.00	
CO	0.46	0.61	0.45	0.84	0.00	0.11	1.00

Supplemental Table 3. Screening, reproductive, and census tract characteristics at baseline and median pollutant values by characteristics.

Variable	*Subjects N=108,002* N (%)	Pollutant Levels at Baseline (1991 Values) Median (25th-75th percentile) PM$_{2.5}$ (μg/m^3)	PM$_{10}$ (μg/m^3)	PM$_{10-2.5}$ (μg/m^3)	NO$_2$ (ppb)	O$_3$ (ppb)	SO$_2$ (ppb)	CO (ppm)
Sigmoidoscopy/Colonoscopy in 1997								
Not Recent	53,673 (49.7)	16.6 (13.5-19.3)	26.2 (23.5-29.5)	10.0 (7.5-12.9)	14.1 (10.3-19.1)	51.6 (44.9-57.6)	6.0 (3.4-7.9)	0.60 (0.46-0.78)
Recent	32,039 (29.7)	16.9 (13.5-19.7)	26.0 (23.4-29.4)	9.9 (7.2-12.8)	15.2 (11.1-19.7)	51.5 (43.9-57.9)	5.7 (3.2-7.9)	0.63 (0.48-0.82)
Unknown	22,290 (20.6)	16.9 (13.7-19.5)	26.4 (23.6-29.9)	10.1 (7.6-13.0)	14.6 (10.7-19.8)	51.8 (45.1-57.8)	6.1 (3.4-8.0)	0.61 (0.47-0.80)
**Men Only**								
PSA Screening in 1997								
Not Recent	8,423 (16.5)	16.4 (13.1-19.3)	26.1 (23.2-29.6)	10.2 (7.7-13.0)	14.3 (10.2-19.3)	50.9 (43.9-56.8)	5.8 (3.3-7.8)	0.61 (0.46-0.80)
Recent	33,329 (65.3)	16.8 (13.7-19.6)	26.1 (23.5-29.4)	9.8 (7.3-12.8)	14.5 (10.7-19.3)	51.9 (45.1-58.0)	6.0 (3.3-7.9)	0.60 (0.47-0.79)
Unknown	9,314 (18.2)	16.8 (13.6-19.5)	26.3 (23.5-30.1)	10.2 (7.7-13.2)	14.9 (10.7-20.0)	51.4 (44.4-57.6)	5.8 (3.2-8.0)	0.62 (0.47-0.83)
**Women Only**								
Mammography								
Not Recent	7,303 (12.8)	16.6 (13.4-19.2)	26.4 (23.6-29.8)	10.2 (7.7-13.1)	14.2 (10.1-19.4)	51.8 (45.6-58.0)	6.3 (3.8-8.1)	0.60 (0.46-0.78)
Recent	49,059 (86.2)	16.8 (13.6-19.5)	26.1 (23.5-29.6)	10.0 (7.4-12.9)	14.6 (10.7-19.4)	51.6 (44.4-57.6)	5.8 (3.2-7.9)	0.61 (0.47-0.80)
Pap Test								
Not Recent	9,060 (15.9)	16.6 (13.5-19.2)	26.3 (23.5-29.8)	10.1 (7.7-12.9)	14.3 (10.3-19.4)	51.6 (45.6-57.7)	6.3 (3.9-8.1)	0.61 (0.46-0.78)
Recent	46,847 (82.3)	16.8 (13.5-19.6)	26.1 (23.5-29.6)	10.0 (7.4-12.9)	14.5 (10.7-19.4)	51.6 (44.3-57.7)	5.8 (3.2-7.9)	0.61 (0.47-0.80)
Hormone Replacement Therapy Use								
Never	23,973 (42.1)	16.7 (13.5-19.3)	26.1 (23.4-29.3)	9.8 (7.4-12.6)	14.6 (10.6-19.7)	52.2 (46.0-58.6)	6.5 (3.9-8.2)	0.60 (0.46-0.78)
Ever ERT	19,207 (33.7)	16.7 (13.4-19.6)	26.3 (23.6-30.0)	10.3 (7.7-13.3)	14.3 (10.4-19.1)	51.0 (43.7-56.5)	5.3 (3.0-7.5)	0.61 (0.47-0.81)
Ever CHRT, No ERT	9,841 (17.3)	16.9 (13.7-19.9)	26.1 (23.6-29.9)	10.0 (7.4-13.1)	14.8 (11.0-19.5)	51.1 (43.9-56.8)	5.2 (2.8-7.5)	0.62 (0.48-0.83)
Other	3,915 (6.9)	16.9 (13.8-19.5)	26.5 (23.7-29.8)	9.9 (7.5-13.0)	14.6 (10.7-19.5)	51.9 (44.6-58.1)	6.1 (3.3-8.0)	0.62 (0.47-0.81)
Number of Live Births and Age at 1st Birth								
Nulliparous	4,308 (7.6)	17.0 (14.0-19.7)	26.4 (23.8-29.8)	10.0 (7.4-12.9)	15.0 (11.1-20.1)	51.2 (44.0-57.5)	5.7 (3.1-7.9)	0.63 (0.48-0.82)
1-2 Births, Age <25	8,931 (15.7)	17.0 (13.8-19.7)	26.4 (23.7-29.9)	10.0 (7.5-13.0)	14.1 (10.3-19.2)	51.9 (44.5-58.0)	5.7 (3.1-7.9)	0.60 (0.46-0.80)
1-2 Births, Age 25+	10,349 (18.2)	17.4 (14.4-20.0)	26.4 (23.8-29.6)	9.6 (7.2-12.6)	15.3 (11.3-20.2)	52.3 (45.5-58.7)	6.2 (3.3-8.1)	0.63 (0.49-0.82)
3+ Births, Age <25	21,785 (38.3)	16.3 (13.0-19.1)	26.1 (23.3-29.6)	10.3 (7.8-13.2)	14.0 (10.1-18.9)	51.2 (44.1-56.7)	5.7 (3.3-7.7)	0.60 (0.45-0.78)
3+ Births, Age 25+	10,153 (17.8)	16.7 (13.6-19.5)	25.9 (23.4-29.3)	9.8 (7.2-12.6)	15.1 (11.1-19.7)	51.5 (44.5-57.9)	6.1 (3.4-8.0)	0.62 (0.48-0.80)

Supplemental Table 3 (continued). Screening, reproductive, and census tract characteristics at baseline and median pollutant values by characteristics.

Variable	*Subjects N=108,002* N (%)	Pollutant Levels at Baseline (1991 Values) Median (25th-75th percentile) PM$_{2.5}$ (μg/m^3)	PM$_{10}$ (μg/m^3)	PM$_{10-2.5}$ (μg/m^3)	NO$_2$ (ppb)	O$_3$ (ppb)	SO$_2$ (ppb)	CO (ppm)
**Census Tract Characteristics**								
Median Household Income								
< $50,000	13,339 (12.4)	15.1 (12.0-18.3)	25.7 (23.0-29.4)	10.7 (8.5-13.6)	10.6 (7.5-14.9)	49.3 (42.1-54.0)	5.1 (3.4-7.2)	0.55 (0.42-0.70)
$50,000-<$100,000	71,153 (65.9)	16.5 (13.3-19.3)	26.3 (23.4-29.7)	10.3 (7.9-13.0)	14.0 (10.5-19.0)	51.2 (43.9-56.8)	5.7 (3.4-7.7)	0.60 (0.46-0.78)
$100,000+	23,510 (21.8)	18.1 (15.9-20.6)	26.2 (24.0-29.4)	8.3 (6.3-11.6)	18.2 (14.4-22.5)	55.6 (49.0-60.6)	7.0 (2.7-8.4)	0.69 (0.53-0.93)
Percent with College Education								
<25%	36,667 (34.0)	16.0 (12.8-18.9)	26.2 (23.3-29.9)	10.7 (8.5-13.4)	12.0 (8.5-16.9)	50.6 (43.8-55.7)	5.4 (3.4-7.6)	0.55 (0.41-0.72)
25-50%	43,480 (40.3)	16.6 (13.5-19.4)	26.2 (23.5-29.7)	10.1 (7.6-13.1)	14.6 (11.1-19.5)	51.8 (44.3-58.0)	6.0 (3.3-7.9)	0.62 (0.48-0.82)
50% or more	27,855 (25.8)	17.7 (15.1-20.4)	26.1 (23.8-29.1)	8.7 (6.3-11.8)	17.6 (13.5-21.4)	52.6 (46.1-59.5)	6.3 (3.1-8.2)	0.68 (0.53-0.91)
Percent African American								
<1%	16,522 (15.3)	14.5 (11.7-17.5)	25.0 (22.0-29.1)	10.5 (8.0-13.7)	9.8 (7.4-16.4)	51.3 (44.9-56.2)	5.5 (3.2-7.3)	0.50 (0.35-0.72)
1%-<10%	68,538 (63.5)	16.7 (13.6-19.5)	26.1 (23.5-29.6)	10.1 (7.5-13.1)	14.0 (11.2-19.7)	51.4 (44.2-58.0)	5.9 (3.2-7.9)	0.63 (0.48-0.84)
10% or More	22,942 (21.2)	18.2 (15.6-20.5)	27.0 (24.7-29.9)	9.4 (7.1-12.1)	15.8 (12.0-19.7)	52.0 (45.6-57.5)	6.2 (3.9-8.2)	0.63 (0.50-0.76)
Percent Other Non-white Race/Ethnicity								
<1%	3,587 (3.3)	16.7 (13.7-18.7)	26.6 (23.7-29.6)	10.2 (8.1-12.5)	9.4 (7.4-13.5)	54.2 (49.4-58.3)	7.7 (5.3-9.9)	0.46 (0.37-0.58)
1%-<10%	69,632 (64.5)	15.9 (12.9-18.6)	25.2 (22.8-28.1)	9.5 (7.3-11.9)	12.8 (9.7-17.0)	51.5 (44.4-57.0)	6.1 (3.9-7.8)	0.55 (0.43-0.69)
10% or More	34,783 (32.2)	18.5 (15.7-21.6)	28.6 (25.4-35.2)	11.7 (8.1-16.5)	19.1 (14.9-24.3)	51.3 (44.4-58.5)	4.8 (2.1-8.0)	0.82 (0.63-1.10)
Unemployment Rate								
<5%	34,293 (31.8)	16.7 (13.4-19.7)	25.9 (23.4-28.7)	9.5 (6.7-12.5)	14.8 (10.9-19.5)	52.2 (44.9-59.0)	6.3 (3.5-8.1)	0.60 (0.47-0.78)
5%-<10%	53,161 (49.2)	16.7 (13.5-19.3)	26.2 (23.4-29.7)	10.0 (7.7-12.9)	14.4 (10.6-19.5)	51.6 (44.5-57.7)	5.9 (3.3-7.9)	0.61 (0.47-0.81)
10% or More	20,548 (19.0)	17.1 (13.9-19.9)	27.0 (23.8-31.4)	10.7 (8.3-13.5)	14.2 (10.0-19.2)	50.8 (43.9-55.3)	5.1 (3.0-7.5)	0.63 (0.46-0.80)
Poverty Rate								
<5%	39,093 (36.2)	17.5 (14.5-19.8)	26.1 (23.5-29.1)	9.00 (6.6-12.0)	16.5 (12.3-20.7)	53.3 (47.5-60.0)	6.7 (3.5-8.2)	0.63 (0.48-0.85)
5%-<10%	32,031 (29.7)	16.6 (13.4-19.4)	26.3 (23.4-29.8)	10.3 (7.7-13.1)	14.2 (10.3-19.4)	51.4 (44.0-57.2)	5.7 (3.1-7.8)	0.60 (0.46-0.79)
10% or More	36,878 (34.1)	16.2 (12.9-19.1)	26.2 (23.6-30.1)	10.7 (8.4-13.4)	12.9 (9.4-17.9)	50.3 (43.0-54.7)	5.1 (3.3-7.4)	0.60 (0.47-0.77)

Supplemental Table 4. Association of PM2.5 with subtypes of cancer by US Region in the CPS-II Nutrition Cohort from 1992-2017

	Northeast		South		Midwest		West		
**Cancer Site**	**Cases**	**PM2.5 by 4.5 μg/m3**	**Cases**	**PM2.5 by 4.5 μg/m3**	**Cases**	**PM2.5 by 4.5 μg/m3**	**Cases**	**PM2.5 by 4.5 μg/m3**	**p-int**
Non-lung Cancer	8,182	0.97 (0.93-1.00)	5,776	0.95 (0.91-0.98)	8,281	0.97 (0.94-1.01)	5,769	1.01 (0.98-1.04)	0.03
Head/Neck	85	0.92 (0.64-1.32)	76	1.01 (0.72-1.42)	92	1.04 (0.75-1.46)	61	1.01 (0.78-1.31)	0.96
Esophagus	75	0.95 (0.64-1.40)	48	0.70 (0.45-1.09)	77	1.26 (0.87-1.85)	64	1.07 (0.83-1.39)	0.21
Stomach	90	0.90 (0.63-1.28)	54	0.80 (0.53-1.20)	93	0.75 (0.53-1.06)	61	1.21 (0.95-1.55)	0.08
Colorectal	787	1.05 (0.94-1.18)	515	0.97 (0.85-1.11)	806	0.94 (0.84-1.06)	528	1.04 (0.95-1.13)	0.45
Liver	43	0.84 (0.49-1.43)	37	0.60 (0.35-1.01)	47	1.82 (1.10-3.01)	28	1.03 (0.71-1.51)	0.02
Gallbladder	17	1.66 (0.76-3.63)	18	1.03 (0.49-2.13)	21	1.01 (0.48-2.11)	8	0.64 (0.28-1.46)	0.43
Pancreas	223	0.91 (0.72-1.14)	168	0.81 (0.64-1.03)	231	0.71 (0.57-0.90)	177	1.01 (0.86-1.19)	0.08
Larynx	45	1.12 (0.69-1.83)	27	0.85 (0.48-1.52)	36	0.81 (0.47-1.38)	26	1.17 (0.79-1.72)	0.63
Melanoma	627	1.06 (0.93-1.21)	451	0.90 (0.78-1.03)	498	0.98 (0.84-1.13)	418	1.02 (0.91-1.13)	0.36
Breast (Invasive)	1,174	0.92 (0.83-1.01)	932	0.93 (0.84-1.02)	1,253	0.91 (0.83-1.00)	895	1.02 (0.95-1.09)	0.12
Breast (In situ)	261	1.02 (0.83-1.25)	191	0.89 (0.72-1.10)	251	0.88 (0.72-1.08)	173	1.05 (0.90-1.23)	0.40
Cervix	8	1.71 (0.55-5.35)	10	0.85 (0.35-2.09)	15	1.14 (0.52-2.53)	8	1.15 (0.63-2.12)	0.82
Uterine	259	0.96 (0.79-1.18)	156	0.97 (0.77-1.22)	257	1.05 (0.86-1.29)	200	1.05 (0.91-1.21)	0.86
Ovarian	112	0.82 (0.60-1.13)	103	0.94 (0.71-1.26)	141	0.92 (0.70-1.20)	91	1.11 (0.90-1.37)	0.40
Prostate	2,166	0.93 (0.87-1.00)	1,486	1.00 (0.93-1.08)	2,210	0.99 (0.92-1.06)	1,458	0.97 (0.92-1.03)	0.49
Bladder	525	0.92 (0.79-1.06)	319	0.82 (0.69-0.98)	457	1.06 (0.91-1.24)	306	0.95 (0.84-1.07)	0.17
Kidney	179	1.14 (0.89-1.46)	125	0.96 (0.73-1.27)	170	1.06 (0.82-1.37)	90	0.99 (0.79-1.23)	0.78
Brain	96	0.92 (0.66-1.28)	54	1.15 (0.77-1.71)	84	0.98 (0.69-1.40)	76	0.84 (0.65-1.09)	0.61
Thyroid	70	0.80 (0.53-1.23)	44	0.93 (0.59-1.49)	59	1.05 (0.68-1.61)	46	0.98 (0.72-1.34)	0.83
Hematologic	885	0.99 (0.88-1.11)	596	0.97 (0.86-1.10)	956	0.91 (0.81-1.01)	691	1.03 (0.95-1.12)	0.34

Stratified on age in 1992, adjusted for gender, race, education, marital status, BMI, smoking status, years smoked, cigarettes/day, years since quit, started smoking <18, years passive smoking, ACS diet score, alcohol, occupational dirtiness, occupational exposure and census tract data (median household income, % college educated, % African American,% Other non-white race, unemployment rate, poverty rate). Female cancers additionally adjusted for: oral contraceptives, HRT Use, age at menarche, age at menopause, age at 1st birth and number of live births. Specific cancer sites with additional adjustment: breast cancer (mammography), cervical cancer (pap test), prostate cancer (PSA test), colorectal (colonoscopy/sigmoidoscopy).

Supplemental Table 5. Alternative covariate models for the association of air pollutants with subtypes of cancer in the CPS-II Nutrition Cohort from 1992-2017.

	Cancer Site	Person-years	Cases	PM2.5 by 4.5 ug/m3	PM10 by 6.7 ug/m3	PM10-2.5 by 5.1 ug/m3	NO2 by 7.2 ppb	Ozone by 9.9 ppb	SO2 by 2.3 ppb	CO by 0.21 ppm
Minimally Adjusted[1]	*Non-Lung Cancers*	1,648,416	28,008	1.00 (0.98-1.02)	0.98 (0.97-0.99)	0.98 (0.96-0.99)	1.01 (1.00-1.02)	0.99 (0.97-1.01)	1.00 (0.98-1.01)	1.01 (1.00-1.03)
	Head/Neck	1,648,416	314	1.05 (0.90-1.23)	0.93 (0.81-1.08)	0.89 (0.77-1.02)	0.95 (0.83-1.09)	0.99 (0.82-1.18)	0.89 (0.78-1.01)	0.98 (0.88-1.10)
	Esophagus	1,648,416	264	1.03 (0.86-1.22)	1.00 (0.86-1.17)	0.99 (0.85-1.14)	1.01 (0.87-1.17)	0.93 (0.76-1.14)	1.03 (0.90-1.18)	1.03 (0.91-1.17)
	Stomach	1,648,416	298	1.00 (0.85-1.17)	1.05 (0.92-1.21)	1.07 (0.93-1.22)	1.03 (0.90-1.18)	0.84 (0.70-1.01)	0.86 (0.75-0.99)	1.03 (0.92-1.16)
	Colorectal	1,648,416	2,636	1.01 (0.96-1.07)	1.05 (1.00-1.10)	1.05 (1.01-1.10)	1.00 (0.95-1.04)	0.99 (0.93-1.05)	1.03 (0.99-1.08)	1.01 (0.97-1.05)
	Liver	1,648,416	155	1.11 (0.88-1.40)	1.01 (0.83-1.23)	0.95 (0.78-1.16)	1.06 (0.87-1.28)	0.86 (0.66-1.12)	1.06 (0.89-1.27)	0.94 (0.78-1.13)
	Gallbladder	1,648,416	64	1.03 (0.73-1.46)	1.08 (0.81-1.44)	1.08 (0.81-1.44)	0.99 (0.74-1.33)	0.92 (0.62-1.38)	1.05 (0.81-1.38)	0.94 (0.72-1.23)
	Pancreas	1,648,416	799	0.92 (0.83-1.02)	1.01 (0.93-1.11)	1.07 (0.98-1.16)	1.05 (0.96-1.14)	0.89 (0.79-1.00)	0.96 (0.88-1.04)	1.07 (0.99-1.15)
	Larynx	1,648,416	134	1.10 (0.87-1.39)	1.06 (0.86-1.29)	1.01 (0.82-1.23)	1.02 (0.84-1.24)	1.04 (0.79-1.37)	1.14 (0.96-1.36)	1.05 (0.89-1.24)
	Melanoma	1,648,416	1,994	1.03 (0.97-1.10)	0.93 (0.88-0.98)	0.89 (0.84-0.95)	1.05 (1.00-1.11)	1.09 (1.01-1.17)	1.00 (0.95-1.05)	1.06 (1.01-1.10)
	Breast (Invasive)	941,561	4,254	0.99 (0.95-1.03)	0.98 (0.94-1.02)	0.98 (0.95-1.02)	1.01 (0.98-1.05)	0.96 (0.92-1.01)	0.97 (0.94-1.00)	1.02 (0.99-1.06)
	Breast (In situ)	941,561	876	1.04 (0.95-1.14)	0.94 (0.86-1.03)	0.90 (0.83-0.98)	1.02 (0.94-1.10)	1.03 (0.92-1.15)	0.97 (0.90-1.04)	1.02 (0.95-1.09)
	Cervix	594,765	41	1.30 (0.87-1.93)	1.40 (1.06-1.85)	1.39 (1.02-1.87)	0.92 (0.65-1.32)	0.82 (0.51-1.31)	0.88 (0.64-1.21)	1.03 (0.78-1.36)
	Uterine	594,765	872	1.03 (0.94-1.14)	1.11 (1.03-1.20)	1.12 (1.04-1.21)	1.05 (0.97-1.13)	1.01 (0.91-1.13)	0.96 (0.89-1.03)	1.07 (1.00-1.14)
	Ovarian	739,916	447	1.00 (0.88-1.14)	0.98 (0.87-1.11)	0.98 (0.87-1.09)	1.00 (0.90-1.12)	0.94 (0.81-1.09)	0.90 (0.81-1.00)	1.00 (0.91-1.10)
	Prostate	706,855	7,320	0.98 (0.95-1.01)	0.97 (0.94-1.00)	0.98 (0.95-1.00)	0.99 (0.96-1.02)	1.02 (0.99-1.06)	1.01 (0.98-1.03)	0.99 (0.97-1.01)
	Bladder	1,648,416	1,607	0.97 (0.91-1.05)	0.89 (0.83-0.95)	0.89 (0.83-0.94)	0.99 (0.93-1.05)	0.92 (0.85-0.99)	1.05 (0.99-1.11)	0.98 (0.93-1.03)
	Kidney	1,648,416	564	1.04 (0.93-1.17)	0.96 (0.86-1.07)	0.92 (0.83-1.02)	1.01 (0.91-1.11)	1.07 (0.93-1.23)	1.11 (1.02-1.22)	1.01 (0.93-1.10)
	Brain	1,648,416	310	0.92 (0.79-1.08)	0.96 (0.84-1.11)	1.01 (0.88-1.15)	1.03 (0.90-1.17)	1.09 (0.91-1.31)	1.02 (0.90-1.15)	1.05 (0.94-1.17)
	Thyroid	1,648,416	219	1.00 (0.83-1.21)	0.98 (0.83-1.16)	0.98 (0.83-1.15)	1.02 (0.87-1.20)	1.17 (0.94-1.47)	0.98 (0.84-1.14)	1.01 (0.87-1.16)
	Hematologic	1,648,416	3,128	0.97 (0.93-1.02)	0.97 (0.92-1.01)	0.98 (0.93-1.02)	1.00 (0.96-1.04)	0.97 (0.91-1.02)	0.97 (0.93-1.01)	1.02 (0.98-1.06)
Full Models (No Ecologic Variables)[2]	*Non-Lung Cancers*	1,648,416	28,008	1.00 (0.98-1.01)	0.98 (0.97-1.00)	0.98 (0.97-1.00)	1.01 (0.99-1.02)	1.00 (0.98-1.02)	1.00 (0.98-1.01)	1.01 (1.00-1.03)
	Head/Neck	1,648,416	314	1.05 (0.90-1.22)	0.94 (0.81-1.09)	0.89 (0.77-1.03)	0.96 (0.84-1.10)	1.01 (0.84-1.21)	0.88 (0.78-1.01)	1.00 (0.89-1.12)
	Esophagus	1,648,416	264	1.04 (0.87-1.23)	1.02 (0.88-1.19)	1.00 (0.86-1.17)	1.04 (0.90-1.21)	0.95 (0.78-1.17)	1.01 (0.88-1.16)	1.07 (0.95-1.22)
	Stomach	1,648,416	298	0.99 (0.84-1.17)	1.05 (0.91-1.20)	1.07 (0.93-1.22)	1.04 (0.91-1.19)	0.85 (0.71-1.02)	0.86 (0.75-0.98)	1.05 (0.93-1.17)
	Colorectal	1,648,416	2,636	1.01 (0.96-1.07)	1.05 (1.00-1.10)	1.05 (1.01-1.10)	1.01 (0.96-1.06)	0.99 (0.93-1.06)	1.02 (0.98-1.07)	1.03 (0.99-1.07)
	Liver	1,648,416	155	1.09 (0.87-1.37)	0.97 (0.79-1.18)	0.91 (0.74-1.11)	1.03 (0.85-1.25)	0.88 (0.68-1.15)	1.08 (0.90-1.29)	0.91 (0.76-1.10)
	Gallbladder	1,648,416	64	1.05 (0.74-1.48)	1.07 (0.80-1.43)	1.06 (0.79-1.42)	1.01 (0.75-1.35)	0.94 (0.63-1.41)	1.07 (0.81-1.41)	0.96 (0.73-1.25)
	Pancreas	1,648,416	799	0.92 (0.83-1.02)	1.02 (0.93-1.11)	1.07 (0.99-1.17)	1.05 (0.96-1.14)	0.89 (0.80-1.00)	0.95 (0.87-1.04)	1.07 (0.99-1.15)
	Larynx	1,648,416	134	1.09 (0.86-1.39)	1.09 (0.89-1.34)	1.06 (0.86-1.31)	1.05 (0.86-1.27)	1.07 (0.81-1.42)	1.11 (0.93-1.32)	1.10 (0.93-1.30)
	Melanoma	1,648,416	1,994	1.04 (0.98-1.11)	0.96 (0.90-1.02)	0.92 (0.87-0.98)	1.05 (0.99-1.11)	1.10 (1.02-1.18)	1.02 (0.97-1.07)	1.05 (1.00-1.10)
	Breast (Invasive)	941,561	4,254	0.98 (0.94-1.02)	0.99 (0.95-1.03)	1.00 (0.96-1.04)	1.00 (0.97-1.04)	0.97 (0.92-1.02)	0.97 (0.94-1.00)	1.02 (0.99-1.05)
	Breast (In situ)	941,561	876	1.02 (0.93-1.12)	0.94 (0.86-1.02)	0.91 (0.84-0.99)	1.00 (0.92-1.08)	1.03 (0.92-1.15)	0.98 (0.91-1.06)	1.00 (0.93-1.07)
	Cervix	594,765	41	1.25 (0.83-1.89)	1.40 (1.04-1.88)	1.40 (1.02-1.94)	0.93 (0.65-1.34)	0.75 (0.47-1.22)	0.76 (0.54-1.07)	1.07 (0.81-1.41)
	Uterine	594,765	872	1.02 (0.93-1.12)	1.07 (0.99-1.16)	1.08 (1.00-1.17)	1.04 (0.97-1.13)	1.02 (0.91-1.13)	0.97 (0.90-1.05)	1.05 (0.99-1.12)
	Ovarian	739,916	447	0.99 (0.87-1.13)	0.98 (0.87-1.10)	0.98 (0.87-1.10)	0.99 (0.88-1.10)	0.94 (0.81-1.09)	0.90 (0.81-1.00)	0.99 (0.90-1.09)
	Prostate	706,855	7,320	0.98 (0.95-1.01)	0.97 (0.95-1.00)	0.98 (0.95-1.01)	0.99 (0.96-1.01)	1.02 (0.98-1.05)	1.01 (0.98-1.03)	0.98 (0.96-1.01)
	Bladder	1,648,416	1,607	0.97 (0.90-1.04)	0.91 (0.85-0.97)	0.91 (0.85-0.97)	0.99 (0.94-1.06)	0.94 (0.87-1.02)	1.04 (0.98-1.10)	0.99 (0.94-1.05)
	Kidney	1,648,416	564	1.05 (0.93-1.18)	0.94 (0.84-1.05)	0.90 (0.81-1.00)	1.01 (0.92-1.12)	1.07 (0.93-1.23)	1.10 (1.00-1.20)	1.02 (0.94-1.12)
	Brain	1,648,416	310	0.92 (0.79-1.08)	0.96 (0.83-1.11)	1.00 (0.88-1.15)	1.03 (0.90-1.17)	1.09 (0.91-1.31)	1.03 (0.91-1.16)	1.04 (0.93-1.16)
	Thyroid	1,648,416	219	1.01 (0.83-1.22)	0.98 (0.83-1.17)	0.97 (0.82-1.15)	1.03 (0.88-1.21)	1.16 (0.93-1.45)	0.98 (0.84-1.15)	1.01 (0.87-1.16)
	Hematologic	1,648,416	3,128	0.97 (0.93-1.03)	0.97 (0.93-1.01)	0.98 (0.94-1.02)	1.00 (0.96-1.04)	0.97 (0.91-1.03)	0.97 (0.93-1.01)	1.02 (0.98-1.06)

1 Stratified on age in 1992 and adjusted for gender

2 Stratified on age in 1992, adjusted for gender, race, education, marital status, BMI, smoking status, years smoked, cigarettes/day, years since quit, started smoking <18, years passive smoking, ACS diet score, alcohol, occupational dirtiness, occupational exposure and census tract data. Female cancers additionally adjusted for: oral contraceptives, HRT Use, age at menarche, age at menopause, age at 1st birth and number of live births. Specific cancer sites with additional adjustment: breast cancer (mammography), cervical cancer (pap test), prostate cancer (PSA test), colorectal (colonoscopy/sigmoidoscopy).

Supplemental Table 6. Alternative exposure timing with 5-year moving averages for the association with air pollutants with subtypes of cancer in the CPS-II Nutrition Cohort from 1992-2017.

Cancer Site	Person-years	Cases	PM2.5 by 4.5 ug/m3	PM10 by 6.7 ug/m3	PM10-2.5 by 5.1 ug/m3	NO2 by 7.2 ppb	Ozone by 9.9 ppb	SO2 by 2.3 ppb	CO by 0.21 ppm
Non-Lung Cancers	1,648,416	28,008	0.99 (0.97-1.01)	0.98 (0.97-1.00)	0.98 (0.97-1.00)	1.00 (0.98-1.01)	0.99 (0.97-1.01)	1.00 (0.99-1.02)	1.01 (0.99-1.02)
Head/Neck	1,648,416	314	1.04 (0.88-1.24)	0.94 (0.81-1.09)	0.90 (0.77-1.04)	0.91 (0.77-1.07)	1.04 (0.86-1.26)	0.86 (0.74-1.00)	0.93 (0.80-1.08)
Esophagus	1,648,416	264	0.98 (0.81-1.20)	0.99 (0.84-1.17)	1.00 (0.85-1.17)	0.99 (0.83-1.18)	0.95 (0.77-1.17)	1.02 (0.87-1.20)	1.04 (0.88-1.23)
Stomach	1,648,416	298	0.99 (0.83-1.19)	0.98 (0.84-1.14)	0.98 (0.84-1.13)	1.00 (0.85-1.17)	0.88 (0.72-1.07)	0.88 (0.75-1.03)	0.99 (0.85-1.15)
Colorectal	1,648,416	2,636	1.02 (0.96-1.08)	1.03 (0.97-1.08)	1.02 (0.97-1.07)	1.01 (0.95-1.06)	1.01 (0.95-1.08)	1.04 (1.00-1.09)	1.04 (0.99-1.09)
Liver	1,648,416	155	1.01 (0.78-1.31)	0.94 (0.76-1.17)	0.92 (0.75-1.14)	1.01 (0.80-1.27)	0.85 (0.65-1.12)	1.06 (0.86-1.32)	0.89 (0.69-1.13)
Gallbladder	1,648,416	64	1.14 (0.76-1.72)	1.29 (0.93-1.78)	1.26 (0.92-1.72)	1.15 (0.79-1.65)	0.87 (0.58-1.33)	1.06 (0.77-1.47)	1.00 (0.69-1.46)
Pancreas	1,648,416	799	0.89 (0.79-1.00)	1.00 (0.91-1.10)	1.07 (0.98-1.17)	1.02 (0.92-1.14)	0.89 (0.79-1.01)	0.95 (0.86-1.05)	1.03 (0.93-1.14)
Larynx	1,648,416	134	0.98 (0.75-1.29)	1.03 (0.82-1.30)	1.05 (0.83-1.32)	0.96 (0.76-1.23)	1.01 (0.75-1.36)	1.03 (0.84-1.28)	1.10 (0.89-1.36)
Melanoma	1,648,416	1,994	1.03 (0.96-1.11)	0.95 (0.90-1.01)	0.92 (0.87-0.98)	1.03 (0.97-1.10)	1.05 (0.97-1.13)	1.04 (0.98-1.10)	1.06 (0.99-1.12)
Breast (Invasive)	941,561	4,254	0.98 (0.93-1.03)	0.99 (0.95-1.03)	1.00 (0.96-1.04)	0.97 (0.93-1.02)	0.97 (0.92-1.02)	0.98 (0.94-1.01)	1.00 (0.96-1.04)
Breast (In situ)	941,561	876	0.98 (0.89-1.09)	0.92 (0.84-1.01)	0.91 (0.83-0.99)	0.97 (0.88-1.06)	1.03 (0.92-1.16)	0.99 (0.91-1.08)	0.95 (0.87-1.04)
Cervix	594,765	41	1.06 (0.69-1.62)	1.21 (0.86-1.70)	1.26 (0.89-1.79)	0.77 (0.51-1.18)	0.78 (0.47-1.31)	0.81 (0.57-1.15)	0.92 (0.65-1.31)
Uterine	594,765	872	1.01 (0.91-1.12)	1.08 (0.99-1.18)	1.10 (1.01-1.20)	1.06 (0.97-1.16)	0.99 (0.88-1.11)	0.97 (0.89-1.05)	1.06 (0.98-1.15)
Ovarian	739,916	447	0.98 (0.85-1.13)	0.98 (0.86-1.11)	0.99 (0.87-1.11)	0.96 (0.84-1.09)	0.92 (0.79-1.08)	0.91 (0.80-1.02)	0.95 (0.84-1.07)
Prostate	706,855	7,320	0.98 (0.95-1.01)	0.98 (0.95-1.01)	0.99 (0.96-1.02)	0.98 (0.95-1.01)	1.01 (0.97-1.05)	1.00 (0.98-1.03)	0.98 (0.95-1.01)
Bladder	1,648,416	1,607	0.94 (0.87-1.02)	0.92 (0.85-0.98)	0.93 (0.87-1.00)	0.97 (0.90-1.04)	0.89 (0.82-0.97)	1.03 (0.96-1.10)	0.99 (0.92-1.06)
Kidney	1,648,416	564	1.07 (0.93-1.22)	0.97 (0.87-1.10)	0.93 (0.83-1.04)	1.07 (0.95-1.21)	1.01 (0.88-1.17)	1.14 (1.02-1.26)	1.10 (0.98-1.24)
Brain	1,648,416	310	0.96 (0.81-1.15)	1.01 (0.86-1.17)	1.03 (0.89-1.19)	1.08 (0.93-1.27)	1.09 (0.90-1.33)	1.05 (0.92-1.21)	1.09 (0.94-1.26)
Thyroid	1,648,416	219	0.97 (0.78-1.20)	1.00 (0.84-1.20)	1.02 (0.86-1.22)	1.01 (0.83-1.22)	1.09 (0.86-1.39)	0.91 (0.76-1.10)	1.03 (0.86-1.24)
Hematologic	1,648,416	3,128	0.99 (0.93-1.05)	0.96 (0.91-1.01)	0.96 (0.91-1.00)	1.01 (0.96-1.06)	0.99 (0.93-1.05)	0.98 (0.93-1.03)	1.02 (0.97-1.08)

Stratified on age in 1992, adjusted for gender, race, education, marital status, BMI, smoking status, years smoked, cigarettes/day, years since quit, started smoking <18, years passive smoking, ACS diet score, alcohol, occupational dirtiness, occupational exposure and census tract data (median household income, % college educated, % African American,% Other non-white race, unemployment rate, poverty rate). Female cancers additionally adjusted for: oral contraceptives, HRT Use, age at menarche, age at menopause, age at 1st birth and number of live births. Specific cancer sites with additional adjustment: breast cancer (mammography), cervical cancer (pap test), prostate cancer (PSA test), colorectal (colonoscopy/sigmoidoscopy).

Supplemental Table 7. Limiting follow-up to the period with updated address history (1997-2017) for the association of air pollutants with subtypes of cancer in the CPS-II Nutrition Cohort.

Cancer Site	Person-years	Cases	PM2.5 by 4.5 ug/m3	PM10 by 6.7 ug/m3	PM10-2.5 by 5.1 ug/m3	NO2 by 7.2 ppb	Ozone by 9.9 ppb	SO2 by 2.3 ppb	CO by 0.21 ppm
Non-Lung Cancers	1,248,815	22,621	0.99 (0.97-1.01)	0.97 (0.95-0.99)	0.97 (0.96-0.99)	1.00 (0.98-1.02)	0.99 (0.97-1.01)	1.00 (0.99-1.02)	1.02 (1.00-1.04)
Head/Neck	1,248,815	245	0.95 (0.77-1.17)	0.88 (0.73-1.06)	0.88 (0.74-1.06)	0.92 (0.77-1.12)	0.94 (0.76-1.15)	0.92 (0.76-1.11)	0.93 (0.76-1.13)
Esophagus	1,248,815	221	1.01 (0.81-1.26)	0.98 (0.81-1.19)	0.97 (0.81-1.17)	1.03 (0.84-1.25)	0.97 (0.77-1.22)	1.13 (0.94-1.35)	1.10 (0.90-1.35)
Stomach	1,248,815	242	1.04 (0.84-1.28)	1.03 (0.86-1.22)	1.01 (0.85-1.20)	1.03 (0.86-1.24)	0.86 (0.69-1.06)	0.94 (0.77-1.14)	0.92 (0.76-1.13)
Colorectal	1,248,815	2,081	1.00 (0.94-1.08)	1.02 (0.96-1.08)	1.02 (0.96-1.08)	1.04 (0.98-1.11)	1.00 (0.93-1.08)	1.06 (1.00-1.13)	1.06 (1.00-1.13)
Liver	1,248,815	133	1.09 (0.82-1.46)	0.90 (0.71-1.16)	0.83 (0.65-1.07)	0.99 (0.77-1.28)	0.86 (0.65-1.15)	1.24 (0.97-1.58)	0.91 (0.68-1.21)
Gallbladder	1,248,815	56	1.27 (0.80-2.01)	1.40 (0.96-2.04)	1.28 (0.90-1.81)	1.24 (0.82-1.88)	0.97 (0.62-1.51)	1.06 (0.73-1.54)	1.16 (0.75-1.79)
Pancreas	1,248,815	689	0.89 (0.78-1.01)	1.00 (0.90-1.11)	1.06 (0.96-1.18)	1.00 (0.89-1.12)	0.96 (0.84-1.09)	0.99 (0.88-1.11)	1.03 (0.91-1.16)
Larynx	1,248,815	106	0.88 (0.64-1.22)	1.00 (0.75-1.32)	1.08 (0.82-1.41)	0.79 (0.59-1.07)	1.03 (0.74-1.42)	1.05 (0.81-1.37)	0.85 (0.63-1.15)
Melanoma	1,248,815	1,717	1.04 (0.96-1.12)	0.97 (0.91-1.04)	0.95 (0.89-1.01)	1.00 (0.93-1.07)	1.05 (0.97-1.14)	0.99 (0.93-1.07)	1.05 (0.97-1.13)
Breast (Invasive)	731,106	3,443	0.94 (0.89-1.00)	0.96 (0.91-1.01)	0.98 (0.94-1.03)	0.97 (0.92-1.02)	0.95 (0.90-1.00)	0.96 (0.92-1.01)	1.03 (0.97-1.08)
Breast (In situ)	731,106	716	1.04 (0.92-1.17)	0.94 (0.85-1.05)	0.91 (0.82-1.00)	0.99 (0.89-1.10)	1.08 (0.95-1.22)	1.02 (0.91-1.13)	1.00 (0.90-1.11)
Cervix	456,392	23	1.19 (0.60-2.38)	1.21 (0.69-2.12)	1.14 (0.65-2.00)	0.69 (0.37-1.30)	0.80 (0.40-1.59)	0.65 (0.34-1.25)	0.80 (0.42-1.50)
Uterine	456,392	670	1.04 (0.92-1.18)	1.03 (0.93-1.15)	1.01 (0.91-1.13)	1.05 (0.94-1.17)	1.04 (0.91-1.18)	1.00 (0.90-1.12)	0.99 (0.89-1.11)
Ovarian	568,280	348	1.01 (0.85-1.20)	0.98 (0.84-1.13)	0.97 (0.84-1.12)	0.99 (0.85-1.16)	0.93 (0.78-1.11)	0.96 (0.82-1.12)	1.01 (0.86-1.17)
Prostate	517,709	5,563	0.99 (0.94-1.03)	0.99 (0.95-1.02)	0.99 (0.96-1.03)	1.00 (0.96-1.04)	1.02 (0.97-1.06)	1.01 (0.97-1.05)	1.00 (0.96-1.04)
Bladder	1,248,815	1,356	0.94 (0.86-1.03)	0.89 (0.82-0.96)	0.90 (0.83-0.97)	0.99 (0.91-1.07)	0.89 (0.81-0.97)	1.06 (0.98-1.15)	1.04 (0.95-1.13)
Kidney	1,248,815	472	1.14 (0.97-1.33)	1.00 (0.87-1.14)	0.93 (0.82-1.06)	1.04 (0.91-1.19)	1.09 (0.93-1.27)	1.05 (0.92-1.20)	1.09 (0.95-1.26)
Brain	1,248,815	231	0.82 (0.66-1.03)	0.93 (0.76-1.13)	1.03 (0.86-1.23)	1.02 (0.83-1.24)	1.02 (0.82-1.27)	0.88 (0.72-1.08)	1.10 (0.90-1.35)
Thyroid	1,248,815	186	0.95 (0.74-1.22)	0.87 (0.69-1.08)	0.87 (0.71-1.07)	0.94 (0.75-1.17)	1.11 (0.86-1.44)	1.02 (0.82-1.26)	0.88 (0.69-1.12)
Hematologic	1,248,815	2,675	0.97 (0.91-1.03)	0.96 (0.91-1.01)	0.97 (0.92-1.02)	1.00 (0.94-1.05)	0.96 (0.90-1.03)	0.97 (0.91-1.02)	1.05 (0.99-1.11)

Stratified on age in 1992, adjusted for gender, race, education, marital status, BMI, smoking status, years smoked, cigarettes/day, years since quit, started smoking <18, years passive smoking, ACS diet score, alcohol, occupational dirtiness, occupational exposure and census tract data (median household income, % college educated, % African American,% Other non-white race, unemployment rate, poverty rate). Female cancers additionally adjusted for: oral contraceptives, HRT Use, age at menarche, age at menopause, age at 1st birth and number of live births. Specific cancer sites with additional adjustment: breast cancer (mammography), cervical cancer (pap test), prostate cancer (PSA test), colorectal (colonoscopy/sigmoidoscopy).
